# Do little interactions get lost in dark random forests?

**DOI:** 10.1186/s12859-016-0995-8

**Published:** 2016-03-31

**Authors:** Marvin N. Wright, Andreas Ziegler, Inke R. König

**Affiliations:** Institut für Medizinische Biometrie und Statistik, Universität zu Lübeck, Universitätsklinikum Schleswig-Holstein, Campus Lübeck, Ratzeburger Allee 160, Lübeck, 23562 Germany; Zentrum für Klinische Studien, Universität zu Lübeck, Universitätsklinikum Schleswig-Holstein, Campus Lübeck, Lübeck, Germany; School of Mathematics, Statistics and Computer Science, University of KwaZulu-Natal, Pietermaritzburg, South Africa

**Keywords:** Random forests, Trees, Variable importance, Gene-gene interactions, Epistasis

## Abstract

**Background:**

Random forests have often been claimed to uncover interaction effects. However, if and how interaction effects can be differentiated from marginal effects remains unclear. In extensive simulation studies, we investigate whether random forest variable importance measures capture or detect gene-gene interactions. With capturing interactions, we define the ability to identify a variable that acts through an interaction with another one, while detection is the ability to identify an interaction effect as such.

**Results:**

Of the single importance measures, the Gini importance captured interaction effects in most of the simulated scenarios, however, they were masked by marginal effects in other variables. With the permutation importance, the proportion of captured interactions was lower in all cases. Pairwise importance measures performed about equal, with a slight advantage for the joint variable importance method. However, the overall fraction of detected interactions was low. In almost all scenarios the detection fraction in a model with only marginal effects was larger than in a model with an interaction effect only.

**Conclusions:**

Random forests are generally capable of capturing gene-gene interactions, but current variable importance measures are unable to detect them as interactions. In most of the cases, interactions are masked by marginal effects and interactions cannot be differentiated from marginal effects. Consequently, caution is warranted when claiming that random forests uncover interactions.

**Electronic supplementary material:**

The online version of this article (doi:10.1186/s12859-016-0995-8) contains supplementary material, which is available to authorized users.

## Background

**Fig. 1:**
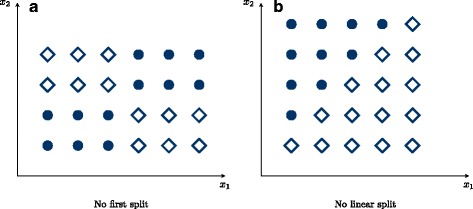
Problematic splits for classification trees and random forests. In (**a**) no reasonable first split on the variables *x*_1_ or *x*_2_ can be made. However, two subsequent splits on *x*_1_ and *x*_2_ split the data perfectly. In (**b**), again no reasonable first split is possible, even though the classes are linear separable. Both the variables *x*_1_ or *x*_2_ have to be considered simultaneously and even with several subsequent splits on *x*_1_ and *x*_2_, no accurate classification is possible

Random forests have often been claimed to uncover interaction effects [1–8]. This is deduced from the recursive structure of trees, which generally enables them to take dependencies into account in a hierarchical manner. Specifically, a different behavior in the two branches after a split indicates possible interactions between the predictor variables [9]. However, some variable combinations without clear marginal effects might make the tree algorithm ineffective (see Fig. 1). In particular in random forests, it is difficult to differentiate between a real interaction effect, marginal effects and just random variations.

To investigate how random forests deal with interaction effects, we are interested in two aspects. For the first, we consider an example reported in the studies by Droździk et al. [10] and Zschiedrich et al. [11] on a polymorphism in the *MDR1* gene as a susceptibility factor for Parkinson’s disease. Only a very small marginal genetic effect was shown, but there was a significant interaction between the variant and pesticide exposure on disease risk. Hence, it is of interest whether this genetic variant would nonetheless be identified as a predictor in random forests. If a variable is identified by the random forest that contributes to the classification with an interaction effect, this interaction effect is *captured* by the model. The second aspect is whether random forests are able to identify the interaction effect per se and the predictor variables interacting with each other. We will use the term *detect* for this in the following. Many authors argue that random forests capture interactions [1–5], while others even state that they identify, uncover or detect them [6–8]. However, empirical studies are rare.

It has been shown that variable importance measures are, in principle, suitable to capture interactions [12, 13]. However, current methods seem to fail in high dimensional data [14], and the effect of various different interaction models on importance measures has not been investigated. To detect interactions, the standard variable importance measures of random forests, Gini and permutation importance, are by design not suitable. Therefore, different methods specifically designed to detect effects of pairs of variables in random forests were proposed [15–17]. These methods measure a joint variable importance to rank variable pairs by their interaction effects. The efficacy of these approaches has only been investigated in small simulations and without considering marginal effects or different interaction scenarios.

In an extensive simulation study, we therefore investigate whether random forests variable importance measures capture or detect interactions effects. In the first part, the Gini and permutation variable importance measures are used to capture interaction effects between single nucleotide polymorphisms (SNPs). Since these methods cannot detect interaction effects, we consider only the pairwise importance measures in the second part, in which we focus on the detection of interacting SNPs. In our simulation, we consider various interaction models, vary effect sizes, minor allele frequencies (MAF) and the number of SNPs randomly selected as splitting candidates (mtry). Even though SNPs are used as predictive variables, all results naturally generalize to other kinds of categorical data.

## Methods

### Random forests

Detailed descriptions of random forests are available in the original [18] and more recent literature [19, 20]. In brief, random forests are ensembles of decision trees. Depending on the outcome, trees can be classification or regression trees (CARTs) [21], survival trees [22] or probability estimation trees (PETs) [23], among others. For random forests, a number of trees are grown that differ because of two components. First, each tree is based on a prespecified number of bootstrap samples or subsamples of individuals. Second, only a random subset of the variables is considered as splitting candidates at each split in the trees. To classify a subject in the random forest, the results of the single trees are aggregated in an appropriate way, depending on the type of random forest. A great advantage of random forests is that the bootstrapping or subsampling for each tree yields subsets of observations, termed out-of-bag (OOB) observations, which are not included in the tree growing process. These are used to estimate the prediction performance or variable importance. There are two specifically important parameters to random forests: The number mtry of randomly selected splitting candidates is usually kept fixed for all splits. In most implementations, the default value for mtry is $\sqrt {p}$, where *p* is the number of variables in the dataset. However, for datasets with a large number of variables, a larger value is required to capture more relevant variables [3]. Typically, mtry is tuned, e.g. by comparing the prediction performance of several values using cross validation. Another important parameter of random forests is the size of single trees. This size is usually controlled by stopping the tree growth if a minimal terminal node size is reached. For regression and survival outcomes, the terminal node size is usually tuned together with the mtry value, while for classification the trees are grown to purity.

### Gini importance

The standard splitting rule in random forests for classification outcomes is to maximize the decrease of impurity that is introduced by a split. For this, the impurity is typically measured by the Gini index [21]. Since a large Gini index suggests a large decrease of impurity, a split with large Gini index can be considered to be important for classification. Thus, the Gini importance for a variable *x*_*i*_ in a tree can be computed by summing the Gini index values of all nodes in which a split on *x*_*i*_ has been conducted. The average of all tree importance values for *x*_*i*_ is then termed Gini importance of the random forest for *x*_*i*_. In our simulation studies, the R package ranger [24] was used to compute the Gini importance.

### Permutation importance

The basic idea of the permutation variable importance approach [18] is to consider a variable important if it has a positive effect on the prediction performance. To evaluate this, a tree is grown in the first step, and the prediction accuracy in the OOB observations is calculated. In the second step, any association between the variable of interest *x*_*i*_ and the outcome is broken by permuting the values of all individuals for *x*_*i*_, and the prediction accuracy is computed again. The difference between the two accuracy values is the permutation importance for *x*_*i*_ from a single tree. The average of all tree importance values in a random forest then gives the random forest permutation importance of this variable. The procedure is repeated for all variables of interest. The package ranger [24] was used in our analyses.

### Pairwise permutation importance (PPI)

To measure the permutation importance for a pair of variables, a modification of the permutation importance was proposed [15]. Instead of permuting a single variable, two variables *x*_*i*_ and *x*_*j*_ are permuted simultaneously. As for the standard permutation importance, the difference in prediction accuracy with and without permutation is computed and used as importance value for the respective pair of variables. This procedure is repeated for all variable pairs of interest. Here, usually, only a subset of the variable pairs is selected to reduce runtime. Although the concept could easily be extended to higher orders of interaction, this would lead to high computational costs. Originally, the approach was implemented in FORTRAN 77 [15]. For easier usage and higher computational speed, we included the PPI measure in the R package ranger [24] (see Additional file 1 for a version including this measure).

### Joint importance by maximal subtrees (JMST)

The joint importance by maximal subtrees measure ([17], JMST) is based on maximal subtrees introduced by Ishwaran et al. [16]. For this, any subtree of the original tree is called an *x*_*i*_-subtree if the root node is split by *x*_*i*_. A subtree is a maximal subtree if it is not a subtree of a larger *x*_*i*_-subtree. It can now be assumed that variables with subtrees closer to the root node have a larger impact on the prediction and are therefore more important than others. The distance of the maximal subtree to the root node is termed the minimal depth of a variable and gives the importance value. For interactions, second-order maximal (*x*_*j*_, *x*_*i*_)-subtrees are used that are defined as the maximal *x*_*j*_-subtree within a maximal *x*_*i*_-subtree. Here, the minimal depth is the distance of the maximal (*x*_*j*_, *x*_*i*_)-subtree to the root of the maximal *x*_*i*_-subtree. For the simulation studies, the find.interaction function of the R package randomForestSRC [25] was used with the option maxsubtree. A matrix with normalized minimal depths for all pairs of variables of interest is returned. Since we are interested in two-way interactions, we used the average of the minimal depths of (*x*_*j*_, *x*_*i*_) and (*x*_*i*_, *x*_*j*_)-subtrees to compute the joint importance of *x*_*i*_ and *x*_*j*_.

### Joint variable importance (JVIMP)

For the joint variable importance measure ([16], JVIMP), maximal subtrees are utilized again. As in permutation importance, the association between a variable *x*_*i*_ and the outcome is broken by randomization. However, instead of permuting the variable, a so-called noise-up procedure is employed: Each observation is dropped down the tree until a maximal *x*_*i*_-subtree is reached. From then on, all further splits are replaced by random child node assignments. This is repeated for all trees. The importance of variable *x*_*i*_ is now measured by the difference between the OOB prediction accuracy of the noised-up forest and the original forest. For pairs of variables *x*_*i*_ and *x*_*j*_, the random assignments start as soon as a maximal subtree of *x*_*i*_ or *x*_*j*_ is reached. The importance of the interaction effect of *x*_*i*_ and *x*_*j*_ is computed by the difference of the sum of both single importance values (additive effect) and the joint importance value (pairwise effect). The find.interaction function of randomForestSRC [25] was used with the option vimp.

### Genetic interaction models

**Fig. 2:**
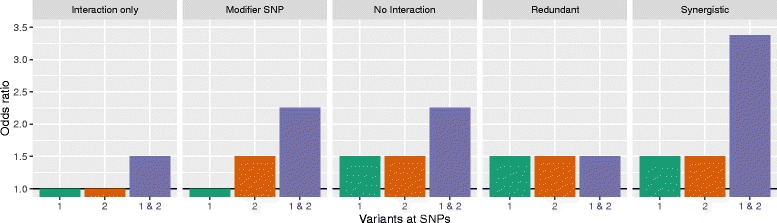
Interaction models. Odds ratios for different interaction models, depending on a variant at the first SNP only, the second SNP only or at both SNPs. If both SNPs have no marginal effect, i.e. an odds ratio of 1, but an effect if variants are present at both SNPs, the model is called *Interaction only*. If only one SNP has a marginal effect and the combined effect is larger than the single marginal effect, the SNP without marginal effect is a *Modifier SNP*. In *No interaction* both SNPs have a marginal effect but no additional combined effect. In this example both SNPs have an odds ratio of 1.5 and the combined effect is exactly 1.5^2^=2.25. If both SNPs have a marginal effect and the combined effect is as large as each single marginal effect, the marginal effects do not add up and we call the model *Redundant*. If both SNPs have a marginal effect and the combined effect is larger than expected by the marginal effects only, it is a *Synergistic* model

We considered two-way interactions between two SNPs based on 5 interaction models. The models were adopted from Lanktree & Hegele [26] but modified for gene-gene instead of gene-environment interactions and illustrated in Fig. 2. First, in *Interaction only*, both SNPs have no marginal effects, i.e. an odds ratio (OR) of 1, but an interaction effect. Second, in *Modifier SNP*, only one SNP has a marginal effect, but the increase for the combination of both is larger than would be expected from marginal effects only. In *No interaction*, both SNPs have marginal effects, but there is no additional interaction effect. In the *Redundant* model, both SNPs have marginal effects, but the combination leads to no further increase in the OR. Finally, in *Synergistic*, both SNPs have a marginal effect and an additional interaction effect in the same direction.

### Simulation studies

Based on these 5 interaction models, data was simulated with varying effect sizes for the interaction effects and marginal effects and different MAF values. In each dataset, two interacting SNPs with marginal and/or interaction effects depending on the interaction model, 5 marginal-only SNPs and 93 noise SNPs were generated. Data was simulated with a sample size of 1000. The phenotypes were simulated with additive effects and logit models, depending on the interaction model (Table 1). The effects were chosen out of *β*_*M*_=(0.4,0.8) and *β*_*I*_=(0.4,0.8), for marginal and interaction effects, respectively. The baseline *β*_0_ was chosen to generate an approximate equal number of cases and controls for each scenario. The MAF was *MAF*_*M*_=(0.2,0.4) and *MAF*_*I*_=(0.2,0.4) for the marginal effect and interaction SNPs, respectively. For the noise SNPs, the MAF was drawn from a uniform distribution between 0 and 1. All simulation parameters are presented in Table 2. The resulting penetrance table for *β*_*I*_=0.4 and *MAF*_*I*_=0.4 is shown in Table 3 for the *Interaction only* model, the penetrance tables for all other interaction models and scenarios are given in the Additional file 2 (Tables S1–S20).

**Table 1:** Logit model generation

Interaction model	SNP1	SNP2	SNP1 x SNP2
Interaction only	0	0	*β*_*I*_
Modifier SNP	0	*β*_*I*_	*β*_*I*_
No interaction	*β*_*I*_	*β*_*I*_	0
Redundant	*β*_*I*_	*β*_*I*_	−*β*_*I*_
Synergistic	*β*_*I*_	*β*_*I*_	*β*_*I*_

In the simulation studies, 2 interacting SNPs and several SNPs having only marginal effects or no effects (noise SNPs) were generated. The phenotypes were simulated with additive effects and logit models. The interacting SNPs have marginal and/or interaction effects, depending on the genetic model. All effects of the interacting SNPs are generated from a single coefficient *β*_*I*_. The table shows marginal effects of SNP1 and SNP2 and the interaction effect. If variants at both SNPs are present, the resulting effect is the sum of the marginal effects and the interaction effect. The baseline *β*_0_ was chosen to generate an approximate equal number of cases and controls for each scenario

**Table 2:** Simulation parameters

Parameter	Description	Values
*β*_*M*_	Effect of marginal-only SNPs	0.4, 0.8
*β*_*I*_	Interaction effect (see Table 1)	0.4, 0.8
*M* *A* *F*_*M*_	Minor allele frequencies for marginal-only SNPs	0.2, 0.4
*M* *A* *F*_*I*_	Minor allele frequencies for interacting SNPs	0.2, 0.4
*mtry*	Number of SNPs randomly selected as splitting candidates	10, 50

All combinations of these parameters were simulated. The interaction models *Interaction only*, *Modifier SNP*, *No interaction*, *Redundant* and *Synergistic* (see Fig. 2) were considered. As variable importance measures, we determined the Gini importance, permutation importance, pairwise permutation importance, joint importance by maximal subtrees and joint variable importance, resulting in a total of 800 simulation scenarios. In addition, one simulation with only 2 marginal-only SNPs and one simulation with 2493 noise SNPs was performed. In both cases, *m* *t* *r* *y*=50, *β*_*M*_=*β*_*I*_=0.4 and *M* *A* *F*_*M*_=*M* *A* *F*_*I*_=0.2 was set. Finally, a simulation with simulated linkage disequilibrium was performed, see the *Methods* section for a description. All simulation scenarios were replicated 100 times

**Table 3:** Penetrance table for model *Interaction only*, *β*_*I*_=0.4, *M* *A* *F*_*I*_=0.4. *A*_1_ and *B*_1_ represent the major alleles and *A*_2_ and *B*_2_ the minor alleles

		SNP 1		
		*A*_1_ *A*_1_	*A*_1_ *A*_2_	*A*_2_ *A*_2_
	*B*_1_ *B*_1_	0.35	0.35	0.35
SNP 2	*B*_1_ *B*_2_	0.35	0.44	0.54
	*B*_2_ *B*_2_	0.35	0.54	0.72

Compared with Table 1 it can be seen that a variant at both SNPs is required for a penetrance larger than the baseline of 0.35. Since the phenotype is simulated with an additive model, the penetrance is increased if two minor alleles are present at one SNP. The penetrances for other models and parameters are computed analogously and are shown in Tables S1–S20 (Additional file 2)

To study the influence of the fixed parameters, we further simulated datasets where the number of marginal-only SNPs was reduced to 2 and datasets where the number of noise SNPs was increased to 2493. In both cases, the effects were fixed to *β*_*M*_=*β*_*I*_=0.4 and the MAF to *MAF*_*M*_=*MAF*_*I*_=0.2. To investigate the impact of linkage disequilibrium (LD), we simulated LD structures based on data from phase 3 of the 1000 genomes project [27]. A random region on chromosome 22 was chosen, and 1000 SNPs without missing data and a MAF between 0.05 and 0.2 were selected. The mean pairwise LD between these SNPs was *D*^′^=0.69 (SD 0.35) and the correlation *r*^2^=0.14 (SD 0.23). For each simulated dataset, 100 SNPs were randomly selected out of these region, and new data with LD structure was simulated using HapSim [28]. Effects of *β*_*M*_=*β*_*I*_=0 and all combinations of *β*_*M*_=(0.4,0.8) and *β*_*I*_=(0.4,0.8) were simulated.

On each dataset, random forests with 500 trees each were grown with a varying number of SNPs randomly selected as splitting candidates (mtry value), chosen from (10,50). To investigate the capture of interacting SNPs, two measures of importance for single variables were computed in the first part, the Gini importance and the permutation importance. Second, to investigate the detection of interactions, we computed the pairwise importance measures PPI [15], JMST [17] and JVIMP [16]. In total, 800 simulation scenarios were analyzed, and for each scenario, we ran 100 replications. Using every importance measure, the variables were ranked, and the ranks of the true interaction SNPs or, in case of the pairwise measures, their combination were saved. Inspired by Lunetta et al. [12] and McKinney et al. [29], the proportion of replicates in which both true interaction SNPs were among the top *k* ranks is plotted for *k*=2,…,10 for the single variable importance measures. A high value for *k*=2 means that the interacting SNPs are ranked before all other SNPs and the interaction is captured by the random forest. High values for *k*=3,…,10 indicate that the interaction is still captured, but masked by marginal effects or noise.

For the pairwise measures, we plot the proportion of replicates in which the combination of the true interacting SNPs was among the top *k* ranks for *k*=1,…,10. To make the analyses computationally feasible, combinations containing noise SNPs were excluded from the ranking. Here, a high value for a small *k* indicates a high proportion of detection of the true interaction, with the exception of the *No interaction* model, where the interaction effect is 0 and any detection would be a false positive result. To compare the ranking of the interacting SNPs with the marginal-only SNPs the proportion of replicates, in which the single variable importance measures ranked the 5 marginal-only SNPs among the top *k* ranks is shown for *k*=5,…,15. For the pairwise importance measures, the proportion of replicates, in which all 10 combinations of marginal-only SNPs are among the top *k* pairs, is shown for *k*=10,…,20. Replication code for all simulation studies is included in Additional file 3.

## Results

### Capturing interaction effects by single variable importance measures

**Fig. 3:**
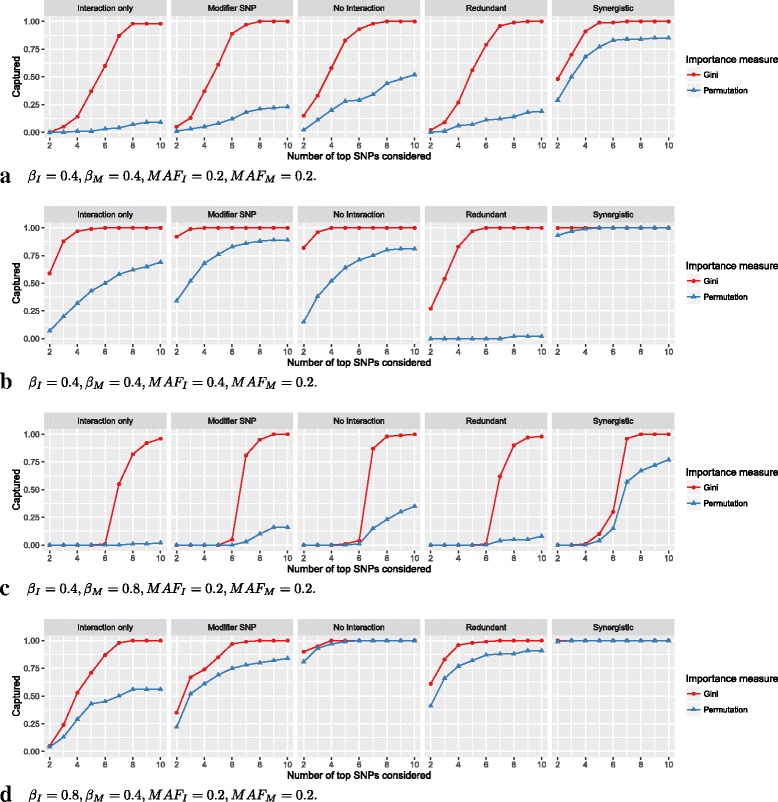
Simulation results for single variable importance measures. On the vertical axis, the proportion of simulation replications in which both interacting SNPs were included in the top *k* SNPs according to the ranking by the variable importance measures is shown. On the horizontal axis, the number of *k* top SNPs considered is shown. If the importance measure included both interacting SNPs in the top *k* SNPs, they were *captured*. In (**a**) to (**d**), different simulation scenarios are shown. The parameters *β*_*I*_ and *β*_*M*_ correspond to the effects of the interacting SNPs and marginal-only SNPs, respectively. *M* *A* *F*_*I*_ and *M* *A* *F*_*M*_ are the minor allele frequencies of the corresponding SNPs. See Figures S1–S24 (Additional file 4) for results of all simulation scenarios for the single variable importance measures

The results for the single variable importance measures and *mtry*=50 are shown in Fig. 3. The Gini importance ranked the interacting SNPs generally higher than the permutation importance. However, the results varied greatly, depending on the interaction model, the simulation scenario and the importance measure. For moderate interaction and marginal effects and equal MAF for interacting and marginal-only SNPs (Fig. 3a), the interacting SNPs were ranked in the top 7 by Gini importance in almost all cases. Comparison with the ranking of marginal-only SNPs (Figure S49, Additional file 4) reveals that most of the other top ranked SNPs were marginal-only. However, some noise SNPs were also included. With permutation importance, the fraction of captured interactions was generally low, except in the *Synergistic* model. Both importance measures achieved a higher capture fraction in *No interaction* than in *Interaction only*, which was expected, since these measures were not designed to detect interactions. When the MAF of the interacting SNPs was increased (Fig. 3b), the capture fraction was higher for both importance measures and all interaction models, except for permutation importance and the *Redundant* model, where the interacting SNPs were almost never ranked in the top 10 SNPs. If instead the MAF of the marginal-only SNPs was increased (Figure S3, Additional file 4), the Gini importance ranked the interacting SNPs between the marginal-only and the noise SNPs in almost all cases. For permutation importance, the results were mostly unchanged. If the effect size of the marginal-only SNPs was increased (Fig. 3c), the Gini importance again ranked the interacting SNPs between the marginal-only and the noise SNPs in almost all cases, while the capture fraction of the permutation importance was very low, except for the *Synergistic* model. If the effect size of the interacting SNPs was increased (Fig. 3d), the capture fraction was generally higher compared with Fig. 3a, in particular for the permutation importance. If MAF and effect sizes were modified at the same time, the described effects added up (Figures S5–S12, Additional file 4). For *mtry*=10, which is the default value in most random forests implementations, the capture fraction was generally lower (Figures S13–S24, Additional file 4). If the number of marginal-only SNPs was reduced to 2 (Figure S97, Additional file 4), the results were mostly similar, except that, as expected, the interacting SNPs were ranked on average 3 ranks higher. If the number of SNPs was increased to 2500 (Figure S98, Additional file 4) and in the case of LD (Figures S99–S103, Additional file 4), the capture fraction was low with both importance measures. In the simulation with LD, the permutation importance ranked the interacting SNPs higher in most of the scenarios.

### Detecting interaction effects by pairwise variable importance measures

**Fig. 4:**
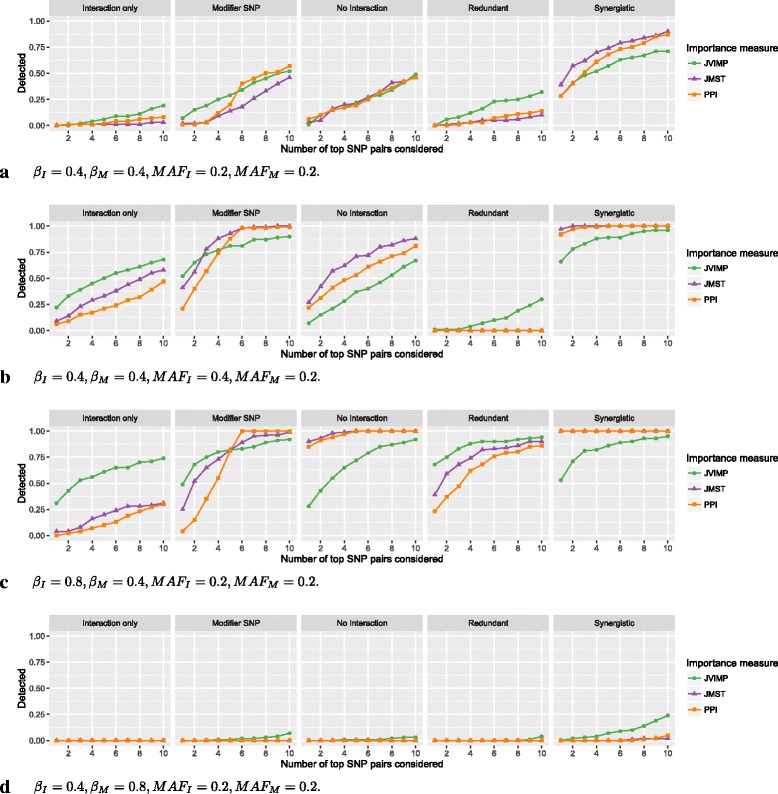
Simulation results for pairwise variable importance measures. On the vertical axis, the proportion of simulation replications in which the true interaction between the two interacting SNPs is included in the top *k* SNP pairs according to the ranking by the variable importance measures is shown. On the horizontal axis, the number of *k* top SNP pairs considered is shown. If the importance measure included the true interaction in the top *k* SNP pairs, the interaction is *detected*. In (**a**) to (**d**), different simulation scenarios are shown. The parameters *β*_*I*_ and *β*_*M*_ correspond to the effects of the interacting SNPs and marginal-only SNPs, respectively. *M* *A* *F*_*I*_ and *M* *A* *F*_*M*_ are the minor allele frequencies of the corresponding SNPs. See Figures S25–S48 (Additional file 4) for results of all simulation scenarios for the single variable importance measures

The results for the pairwise variable importance measures and *mtry*=50 are shown in Fig. 4. The detection fraction was low in all models. The difference between the methods were generally smaller than for the single variable importance measures. For moderate interaction and marginal effects and equal MAF for interacting and marginal-only SNPs (Fig. 4a), the importance measures were about equal, except for *Redundant*, where JVIMP was slightly higher, and for *Synergistic*, where it was lower. Remarkably, with all importance measures, the detection in *No interaction* was higher than in *Interaction only*. When the MAF of the interacting SNPs was increased (Fig. 4b), the detection increased for all models, except for *Redundant*, where it was lower for JMST and PPI and unchanged for JVIMP. In *Interaction only*, the increase was largest, and for JVIMP, the detection was higher than in *No interaction*. If instead the MAF of the marginal-only SNPs was increased (Figure S27, Additional file 4), the detection was slightly lower than in Fig. 4a, in particular for JVIMP. If the effect size of the interacting SNPs was increased (Fig. 4c), the detection was higher for all importance measures. The detection of JVIMP was high for *Interaction only* and low for *No interaction* and *Synergistic*. If the effect size of the marginal-only SNPs was increased (Fig. 4d), the detection was very low in all cases. If MAF and effect sizes were modified at the same time, the described effects added up (Figures S29–S36, Additional file 4). Again, for *mtry*=10, the detection fraction was generally lower (Figures S37–S48, Additional file 4). If the number of marginal-only SNPs was reduced to 2 (Figure S104, Additional file 4), the results were similar for small values of *k*, and the detection was higher for larger values of *k*. This was expected, since combinations including noise variables are excluded in the pairwise measures and thus only 6 combinations of SNPs are possible in this case. If the number of SNPs was increased to 2500 (Figure S105, Additional file 4), the results were comparable to the simulation with 100 SNPs. In the case of LD (Figures S106–S110, Additional file 4), the detection fraction for larger values of *k* was slightly increased. However, this was also the case if no interaction or marginal effects were included, indicating that correlations were detected as interactions.

## Discussion

In our extensive simulation studies, we found that random forests are capable of capturing SNP-SNP interactions, i.e. of including them in the model. Of the single variable importance measures, the Gini importance ranks the interacting SNPs higher than the permutation importance. The single importance measures are unable to detect interactions, and this by design. They can thus not differentiate between marginal and interaction effects. But since, in most cases, the interacting SNPs are ranked higher than noise SNPs even if no marginal effects are present, we conclude that the interaction effects are thereby captured in random forests. In general, the ranking depends heavily on the MAF, with more frequent SNPs being ranked higher.

The results of the pairwise importance measures suggest that they are unable to detect interactions in the presence of marginal effects. With all measures, marginal effects were detected as interaction effects, and true interactions were not found if other SNPs with strong marginal effects were present. Again, SNPs were ranked higher if the MAF was increased. All methods ranked the interacting SNPs higher in the model without interaction, compared with the model with interaction only, suggesting that interaction effects cannot be differentiated from marginal effect. Of the compared methods, JVIMP [16] achieved the best results, since detection was highest for the model with interaction only and lowest for the model without interaction in most of the simulation scenarios.

To study SNP-SNP interactions with random forest, we used 5 interaction models in a simplified setting. We simulated rather strong effects and large MAF values. Our results show that the pairwise importance measures are unable to detect interactions in this setting. In simulations with an increased number of noise SNPs, the single importance measures performed worse and the pairwise measures about equal. If LD was considered, only very strong effects were detected at all and again marginal effects were detected as interactions.

Despite the difficulty of the pairwise variable importance measures to detect interactions, our data suggest that interaction effects are generally captured by random forests. One approach to improve the detection rate might be to use random forests to perform a variable selection first and applying another method to identify interactions subsequently [30, 31]. However, interaction effects might again be masked by marginal effects in that approach. A related idea is to uncover marginal effects in a first step and project the remaining effects on a space orthogonal to the marginal effects, to detect interactions in a second step [32, 33]. On a different route, the detection of interactions might be facilitated by developing new pairwise importance measures based on standard random forests [34]. However, it is argued that in the case of many predictor variables, it is unlikely that interacting variables are selected simultaneously in a given tree [9]. Second, for combinations of variables, the attributable risk [35] can be small, in particular for variants with small MAF. This means that only a small proportion of cases is attributable to the interaction, and even for large effect sizes these interactions are difficult to detect. Finally, it can be argued that random forests are by design unable to split on interactions [36]. As shown in Fig. 1a, if interacting variables have no marginal effect at all, no first split is possible and the interaction cannot be captured. To solve this, the tree growing process in the random forest itself could be modified to better incorporate interactions. A promising, yet computationally intensive new approach are reinforcement learning trees [37], which employ reinforcement learning in each node, to additionally incorporate future splits down in the tree. Several other approaches have been proposed [38], but these are based on single trees only, limiting their usage to low dimensional settings. With fast random forest implementations now available for large sample sizes [39] and high dimensional data [24] these or new methods could be integrated into the random forest framework.

## Conclusions

We conclude that random forests are generally capable of capturing SNP-SNP interactions, but current variable importance measures are unable to detect them. The Gini importance performs better than the permutation importance in identifying SNPs involved in an interaction. However, both methods are not designed to uncover interactions as such, and consequently, in most of the cases, the interactions are masked by other SNPs with marginal effects. None of the pairwise importance measures is able to reliably detect interactions. Marginal effects are detected as interaction effects and here, too, other SNPs with marginal effects hinder the detection of interactions. As a result one should be cautious when claiming that random forests uncover interaction effects.

## Availability of data and materials

The software and simulation code supporting the conclusions of this article are included within the article and its additional files.

## Additional files

Additional file 1 Software package. Version of the software package ranger, including the pairwise permutation importance measure. (GZ 39.9 kb)

Additional file 2 Supplementary tables. Penetrance tables for all genetic interaction models, effect sizes and minor allele frequencies. (PDF 78.1 kb)

Additional file 3 Simulation code. Replication material for all simulation studies. (ZIP 44 kb)

Additional file 4 Supplementary figures. All results for the single and pairwise variable importance measures. (PDF 1105 kb)
