# Supplementary material for: Do little interactions get lost in dark random forests?
Source: BMC Bioinformatics. 2016 Mar 31;17:145. doi: 10.1186/s12859-016-0995-8 (PMC4815164; doi:10.1186/s12859-016-0995-8)
Supplement: Additional file 2 — Supplementary tables. Penetrance tables for all genetic interaction models, effect sizes and minor allele frequencies. (PDF 78.1 kb) [file 12859_2016_995_MOESM2_ESM.pdf]

Supplement for: *Do little interactions get lost in dark random forests?*

Part 1: Penetrance tables

Marvin N. Wright, Andreas Ziegler, Inke R. König

Table S1: Penetrance table for model *no interaction*, $\beta_{\text{I}} = 0.4$, $\text{MAF}_{\text{I}} = 0.2$.

	A_1A_1	A_1A_2	A_2A_2
B_1B_1	0.42	0.52	0.62
B_1B_2	0.52	0.62	0.71
B_2B_2	0.62	0.71	0.78

Table S2: Penetrance table for model *no interaction*, $\beta_{\text{I}} = 0.4$, $\text{MAF}_{\text{I}} = 0.4$.

	A_1A_1	A_1A_2	A_2A_2
B_1B_1	0.35	0.44	0.54
B_1B_2	0.44	0.54	0.64
B_2B_2	0.54	0.64	0.72

Table S3: Penetrance table for model *no interaction*, $\beta_{\text{I}} = 0.8$, $\text{MAF}_{\text{I}} = 0.2$.

	A_1A_1	A_1A_2	A_2A_2
B_1B_1	0.35	0.54	0.72
B_1B_2	0.54	0.72	0.85
B_2B_2	0.72	0.85	0.93

Table S4: Penetrance table for model *no interaction*, $\beta_{\text{I}} = 0.8$, $\text{MAF}_{\text{I}} = 0.4$.

	A_1A_1	A_1A_2	A_2A_2
B_1B_1	0.22	0.38	0.58
B_1B_2	0.38	0.58	0.75
B_2B_2	0.58	0.75	0.87

Table S5: Penetrance table for model *synergistic*, $\beta_{\text{I}} = 0.4$, $\text{MAF}_{\text{I}} = 0.2$.

	A_1A_1	A_1A_2	A_2A_2
B_1B_1	0.42	0.52	0.62
B_1B_2	0.52	0.71	0.84
B_2B_2	0.62	0.84	0.95

Table S6: Penetrance table for model *synergistic*, $\beta_{\mathrm{I}} = 0.4$, $\mathrm{MAF}_{\mathrm{I}} = 0.4$.

	A_1A_1	A_1A_2	A_2A_2
B_1B_1	0.35	0.44	0.54
B_1B_2	0.44	0.64	0.80
B_2B_2	0.54	0.80	0.93

Table S7: Penetrance table for model *synergistic*, $\beta_{\mathrm{I}} = 0.8$, $\mathrm{MAF}_{\mathrm{I}} = 0.2$.

	A_1A_1	A_1A_2	A_2A_2
B_1B_1	0.35	0.54	0.72
B_1B_2	0.54	0.85	0.97
B_2B_2	0.72	0.97	1.00

Table S8: Penetrance table for model *synergistic*, $\beta_{\mathrm{I}} = 0.8$, $\mathrm{MAF}_{\mathrm{I}} = 0.4$.

	A_1A_1	A_1A_2	A_2A_2
B_1B_1	0.22	0.38	0.58
B_1B_2	0.38	0.75	0.94
B_2B_2	0.58	0.94	0.99

Table S9: Penetrance table for model *interaction only*, $\beta_{\mathrm{I}} = 0.4$, $\mathrm{MAF}_{\mathrm{I}} = 0.2$.

	A_1A_1	A_1A_2	A_2A_2
B_1B_1	0.42	0.42	0.42
B_1B_2	0.42	0.52	0.62
B_2B_2	0.42	0.62	0.78

Table S10: Penetrance table for model *interaction only*, $\beta_{\mathrm{I}} = 0.4$, $\mathrm{MAF}_{\mathrm{I}} = 0.4$.

	A_1A_1	A_1A_2	A_2A_2
B_1B_1	0.35	0.35	0.35
B_1B_2	0.35	0.44	0.54
B_2B_2	0.35	0.54	0.72

Table S11: Penetrance table for model *interaction only*, $\beta_{\mathrm{I}} = 0.8$, $\mathrm{MAF_I} = 0.2$.

	A_1A_1	A_1A_2	A_2A_2
B_1B_1	0.35	0.35	0.35
B_1B_2	0.35	0.54	0.72
B_2B_2	0.35	0.72	0.93

Table S12: Penetrance table for model *interaction only*, $\beta_{\mathrm{I}} = 0.8$, $\mathrm{MAF_I} = 0.4$.

	A_1A_1	A_1A_2	A_2A_2
B_1B_1	0.22	0.22	0.22
B_1B_2	0.22	0.38	0.58
B_2B_2	0.22	0.58	0.87

Table S13: Penetrance table for model *modifier snp1*, $\beta_{\mathrm{I}} = 0.4$, $\mathrm{MAF_I} = 0.2$.

	A_1A_1	A_1A_2	A_2A_2
B_1B_1	0.42	0.42	0.42
B_1B_2	0.52	0.62	0.71
B_2B_2	0.62	0.78	0.89

Table S14: Penetrance table for model *modifier snp1*, $\beta_{\mathrm{I}} = 0.4$, $\mathrm{MAF_I} = 0.4$.

	A_1A_1	A_1A_2	A_2A_2
B_1B_1	0.35	0.35	0.35
B_1B_2	0.44	0.54	0.64
B_2B_2	0.54	0.72	0.85

Table S15: Penetrance table for model *modifier snp1*, $\beta_{\mathrm{I}} = 0.8$, $\mathrm{MAF_I} = 0.2$.

	A_1A_1	A_1A_2	A_2A_2
B_1B_1	0.35	0.35	0.35
B_1B_2	0.54	0.72	0.85
B_2B_2	0.72	0.93	0.98

Table S16: Penetrance table for model *modifier snp1*, $\beta_{\mathrm{I}} = 0.8$, $\mathrm{MAF}_{\mathrm{I}} = 0.4$.

	A_1A_1	A_1A_2	A_2A_2
B_1B_1	0.22	0.22	0.22
B_1B_2	0.38	0.58	0.75
B_2B_2	0.58	0.87	0.97

Table S17: Penetrance table for model *redundant*, $\beta_{\mathrm{I}} = 0.4$, $\mathrm{MAF}_{\mathrm{I}} = 0.2$.

	A_1A_1	A_1A_2	A_2A_2
B_1B_1	0.42	0.52	0.62
B_1B_2	0.52	0.52	0.52
B_2B_2	0.62	0.52	0.42

Table S18: Penetrance table for model *redundant*, $\beta_{\mathrm{I}} = 0.4$, $\mathrm{MAF}_{\mathrm{I}} = 0.4$.

	A_1A_1	A_1A_2	A_2A_2
B_1B_1	0.35	0.44	0.54
B_1B_2	0.44	0.44	0.44
B_2B_2	0.54	0.44	0.35

Table S19: Penetrance table for model *redundant*, $\beta_{\mathrm{I}} = 0.8$, $\mathrm{MAF}_{\mathrm{I}} = 0.2$.

	A_1A_1	A_1A_2	A_2A_2
B_1B_1	0.35	0.54	0.72
B_1B_2	0.54	0.54	0.54
B_2B_2	0.72	0.54	0.35

Table S20: Penetrance table for model *redundant*, $\beta_{\mathrm{I}} = 0.8$, $\mathrm{MAF}_{\mathrm{I}} = 0.4$.

	A_1A_1	A_1A_2	A_2A_2
B_1B_1	0.22	0.38	0.58
B_1B_2	0.38	0.38	0.38
B_2B_2	0.58	0.38	0.22
