# Supplementary material for: Do little interactions get lost in dark random forests?
Source: BMC Bioinformatics. 2016 Mar 31;17:145. doi: 10.1186/s12859-016-0995-8 (PMC4815164; doi:10.1186/s12859-016-0995-8)
Supplement: Additional file 4 — Supplementary figures. All results for the single and pairwise variable importance measures. (PDF 1105 kb) [file 12859_2016_995_MOESM4_ESM.pdf]

Supplement for: *Do little interactions get lost in dark random forests?*

Part 2: Single variable importance measures

Marvin N. Wright, Andreas Ziegler, Inke R. König

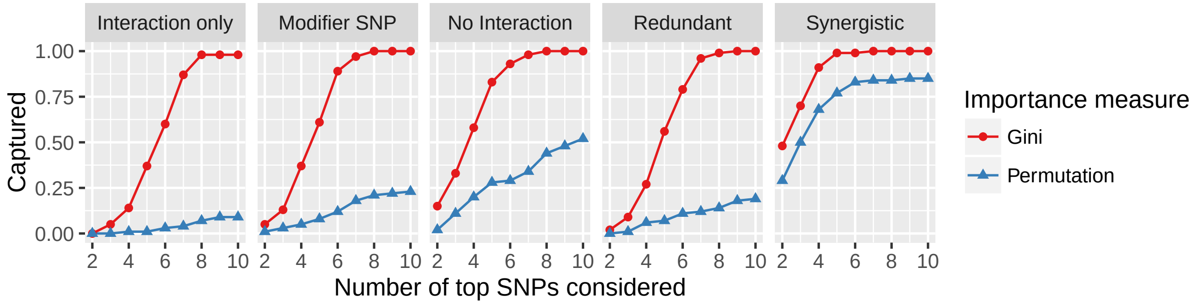

Figure S1: $\beta_I = 0.4, \beta_M = 0.4, MAF_I = 0.2, MAF_M = 0.2, mtry = 50$

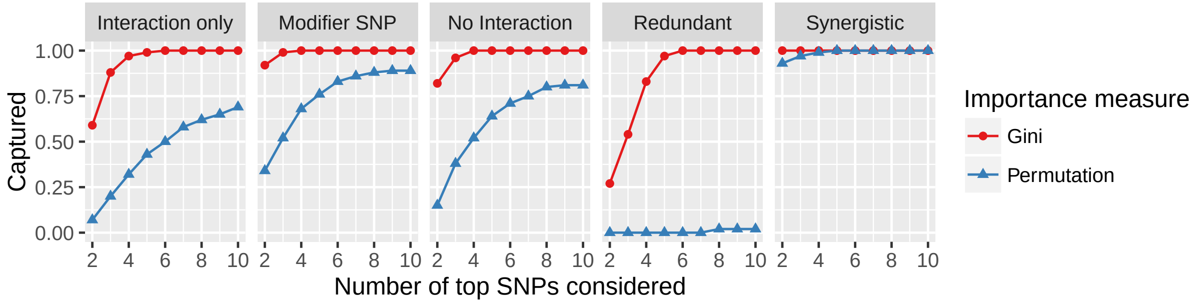

Figure S2: $\beta_I = 0.4, \beta_M = 0.4, MAF_I = 0.4, MAF_M = 0.2, mtry = 50$

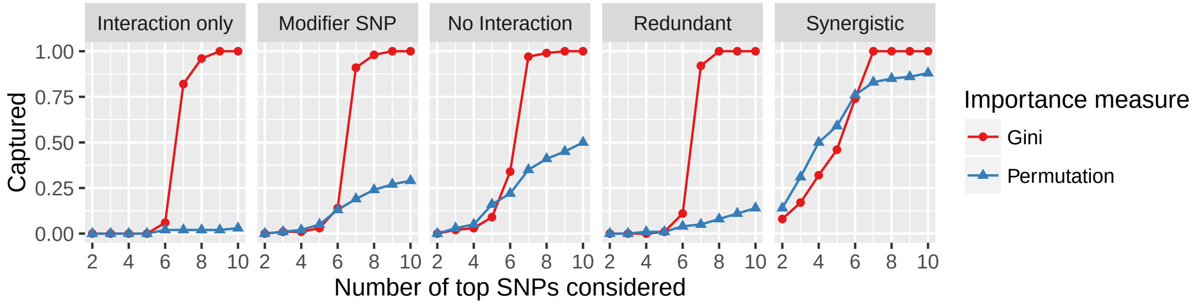

Figure S3: $\beta_I = 0.4, \beta_M = 0.4, MAF_I = 0.2, MAF_M = 0.4, mtry = 50$

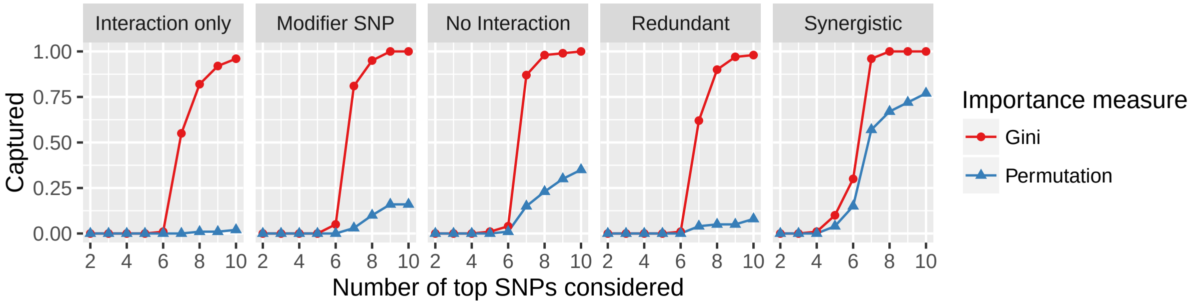

Figure S4: $\beta_I = 0.4, \beta_M = 0.8, MAF_I = 0.2, MAF_M = 0.2, mtry = 50$

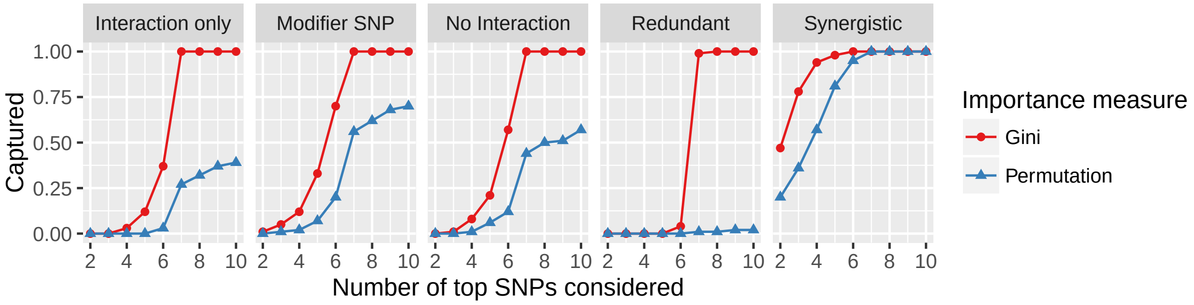

Figure S5: $\beta_I = 0.4, \beta_M = 0.8, MAF_I = 0.4, MAF_M = 0.2, mtry = 50$

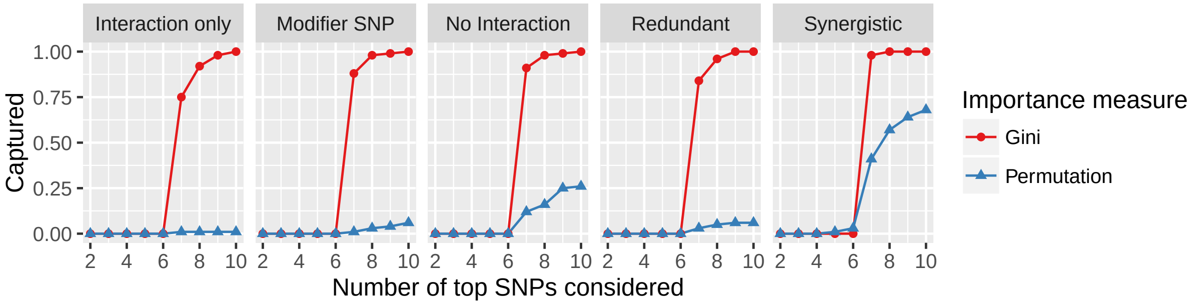

Figure S6: $\beta_I = 0.4, \beta_M = 0.8, MAF_I = 0.2, MAF_M = 0.4, mtry = 50$

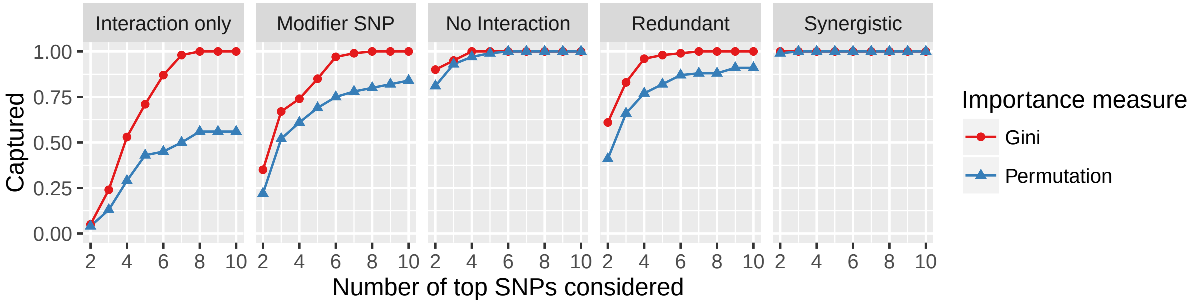

Figure S7: $\beta_I = 0.8, \beta_M = 0.4, MAF_I = 0.2, MAF_M = 0.2, mtry = 50$

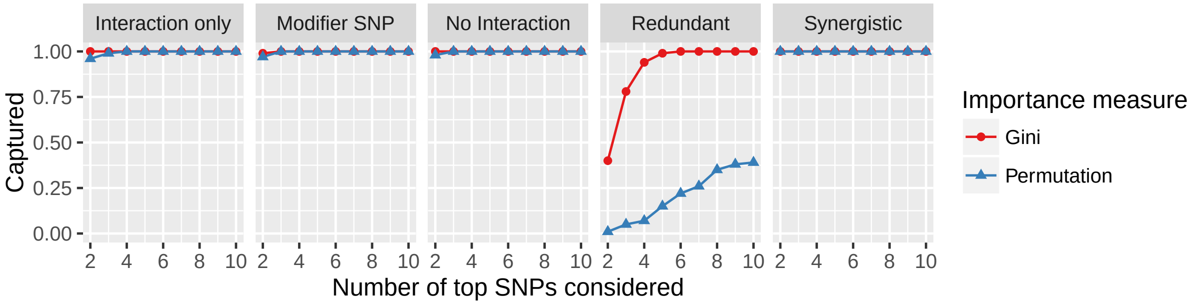

Figure S8: $\beta_I = 0.8, \beta_M = 0.4, MAF_I = 0.4, MAF_M = 0.2, mtry = 50$

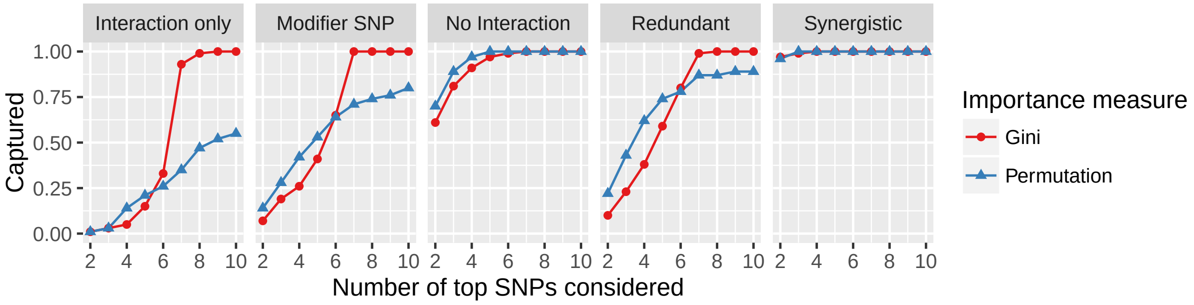

Figure S9: $\beta_I = 0.8, \beta_M = 0.4, MAF_I = 0.2, MAF_M = 0.4, mtry = 50$

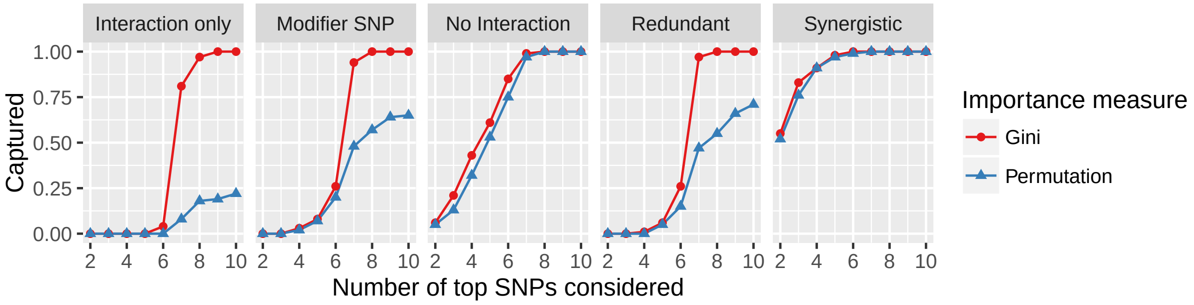

Figure S10: $\beta_I = 0.8, \beta_M = 0.8, MAF_I = 0.2, MAF_M = 0.2, mtry = 50$

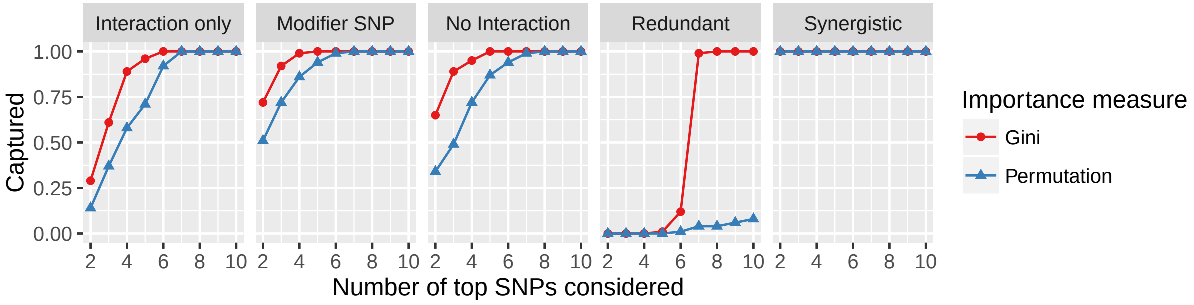

Figure S11: $\beta_I = 0.8, \beta_M = 0.8, MAF_I = 0.4, MAF_M = 0.2, mtry = 50$

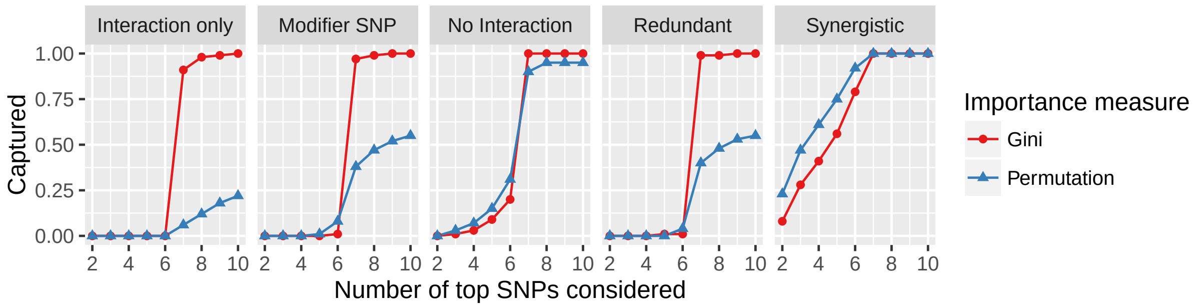

Figure S12: $\beta_I = 0.8, \beta_M = 0.8, MAF_I = 0.2, MAF_M = 0.4, mtry = 50$

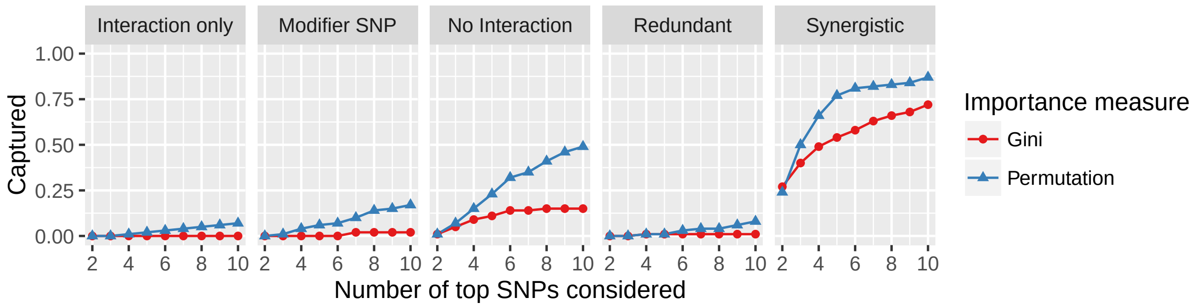

Figure S13: $\beta_I = 0.4, \beta_M = 0.4, MAF_I = 0.2, MAF_M = 0.2, mtry = 10$

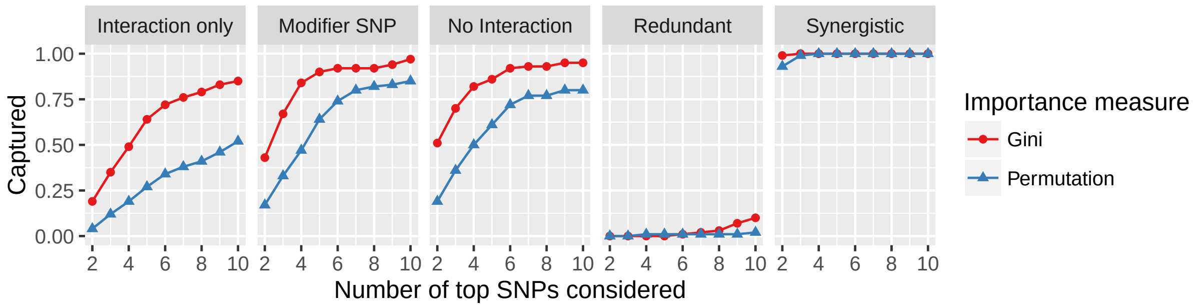

Figure S14: $\beta_I = 0.4, \beta_M = 0.4, MAF_I = 0.4, MAF_M = 0.2, mtry = 10$

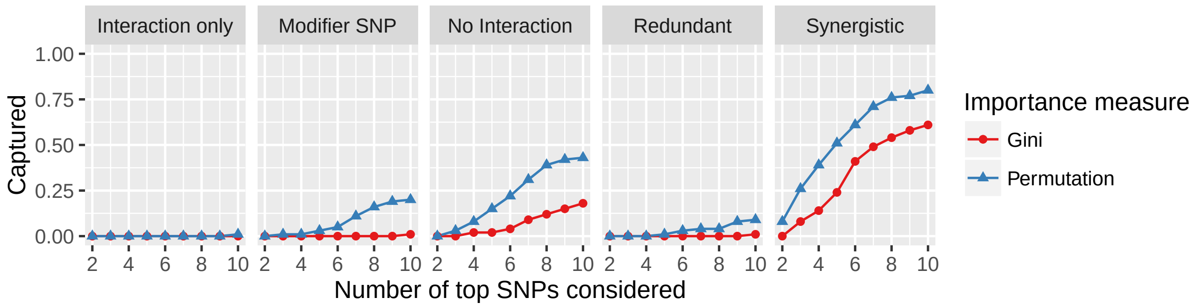

Figure S15: $\beta_I = 0.4, \beta_M = 0.4, MAF_I = 0.2, MAF_M = 0.4, mtry = 10$

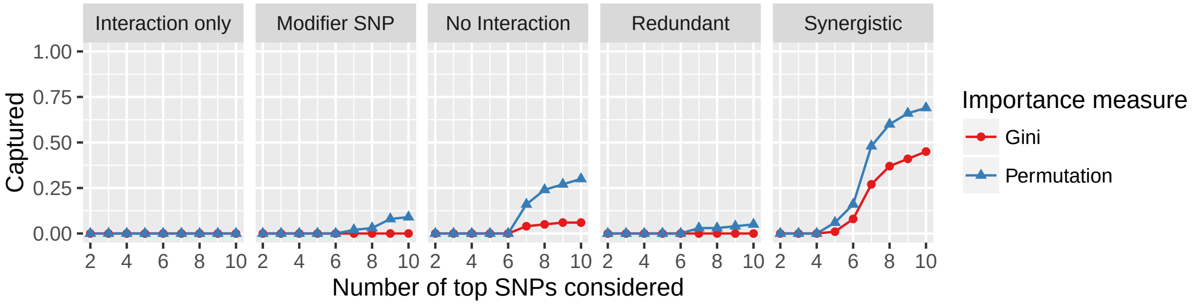

Figure S16: $\beta_I = 0.4, \beta_M = 0.8, MAF_I = 0.2, MAF_M = 0.2, mtry = 10$

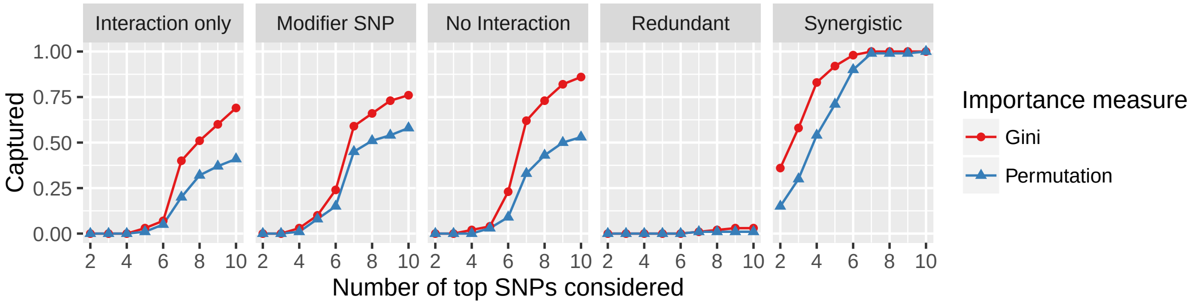

Figure S17: $\beta_I = 0.4, \beta_M = 0.8, MAF_I = 0.4, MAF_M = 0.2, mtry = 10$

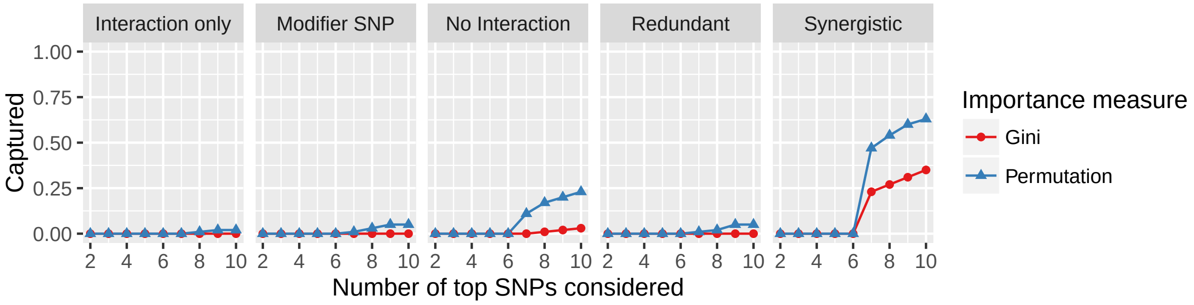

Figure S18: $\beta_I = 0.4, \beta_M = 0.8, MAF_I = 0.2, MAF_M = 0.4, mtry = 10$

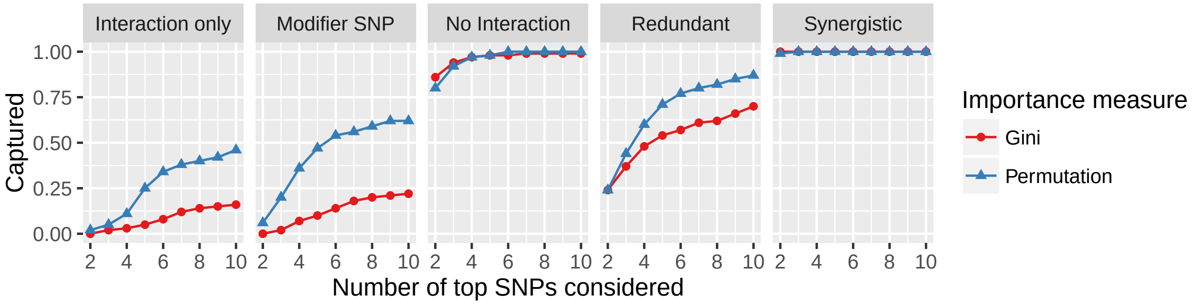

Figure S19: $\beta_I = 0.8, \beta_M = 0.4, MAF_I = 0.2, MAF_M = 0.2, mtry = 10$

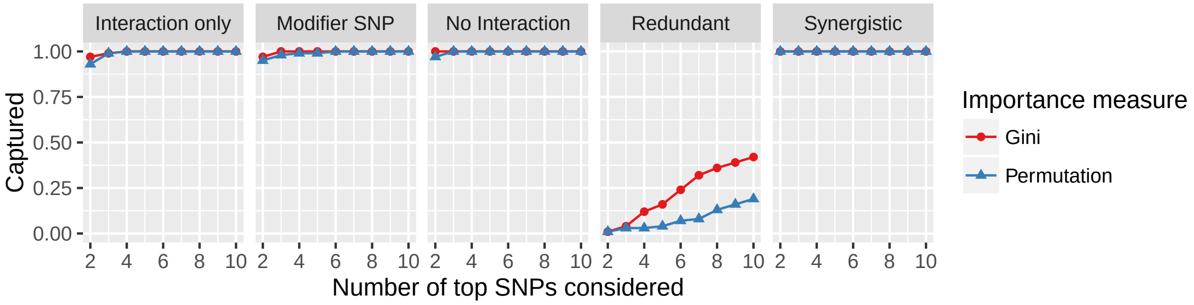

Figure S20: $\beta_I = 0.8, \beta_M = 0.4, MAF_I = 0.4, MAF_M = 0.2, mtry = 10$

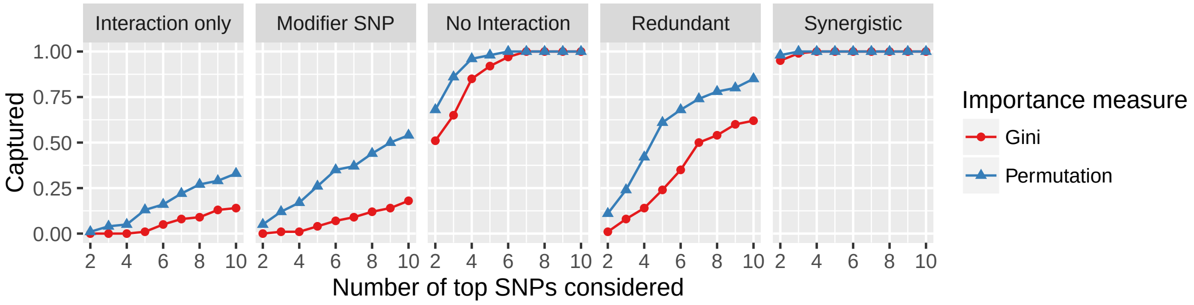

Figure S21: $\beta_I = 0.8, \beta_M = 0.4, MAF_I = 0.2, MAF_M = 0.4, mtry = 10$

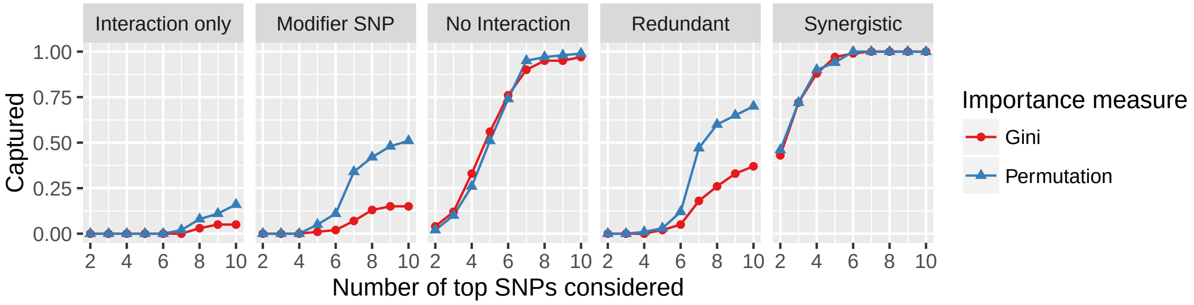

Figure S22: $\beta_I = 0.8, \beta_M = 0.8, MAF_I = 0.2, MAF_M = 0.2, mtry = 10$

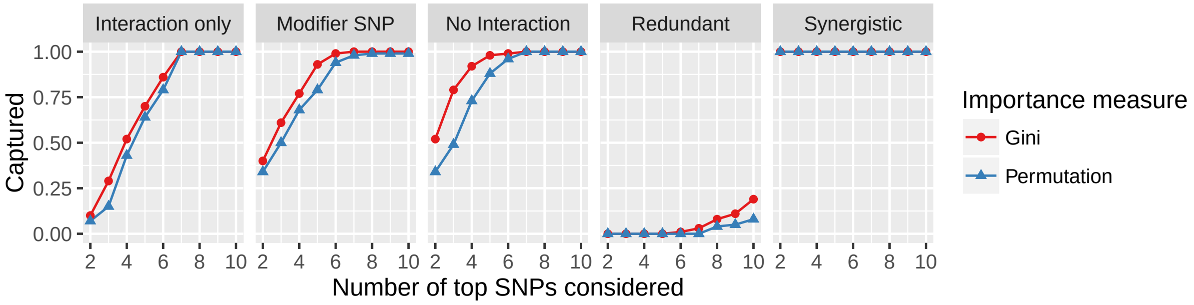

Figure S23: $\beta_I = 0.8, \beta_M = 0.8, MAF_I = 0.4, MAF_M = 0.2, mtry = 10$

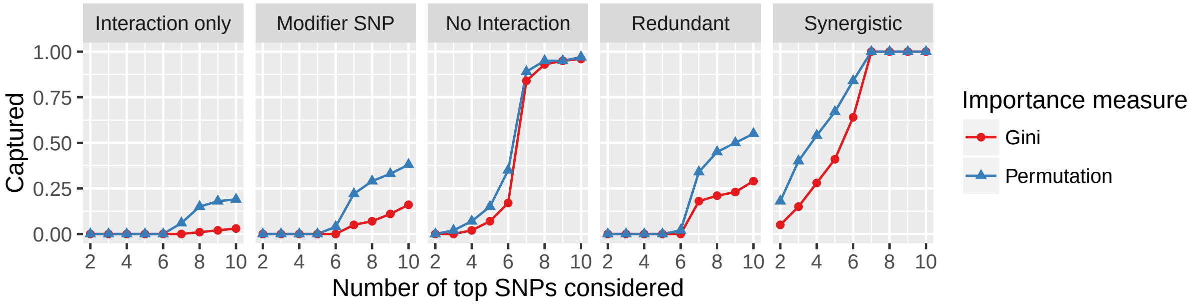

Figure S24: $\beta_I = 0.8, \beta_M = 0.8, MAF_I = 0.2, MAF_M = 0.4, mtry = 10$

Supplement for: *Do little interactions get lost in dark random forests?*

Part 3: Pairwise variable importance measures

Marvin N. Wright, Andreas Ziegler, Inke R. König

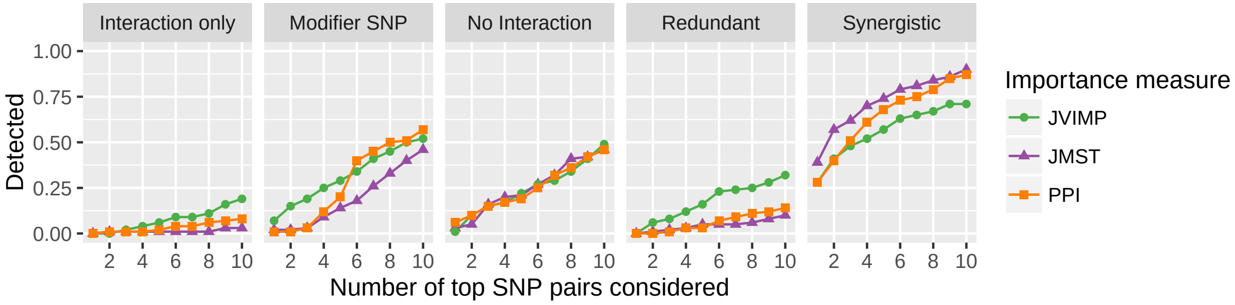

Figure S25: $\beta_I = 0.4, \beta_M = 0.4, MAF_I = 0.2, MAF_M = 0.2, mtry = 50$

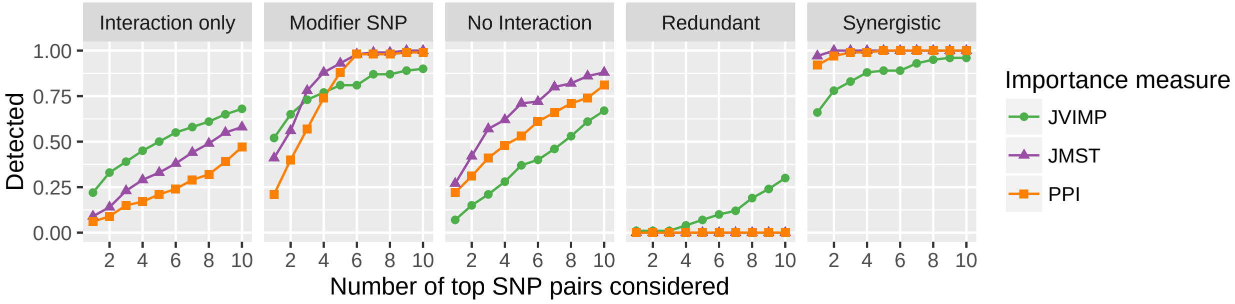

Figure S26: $\beta_I = 0.4, \beta_M = 0.4, MAF_I = 0.4, MAF_M = 0.2, mtry = 50$

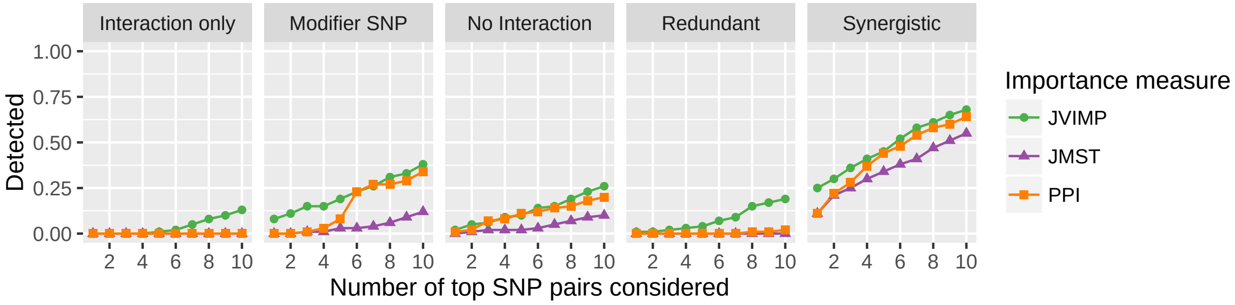

Figure S27: $\beta_I = 0.4, \beta_M = 0.4, MAF_I = 0.2, MAF_M = 0.4, mtry = 50$

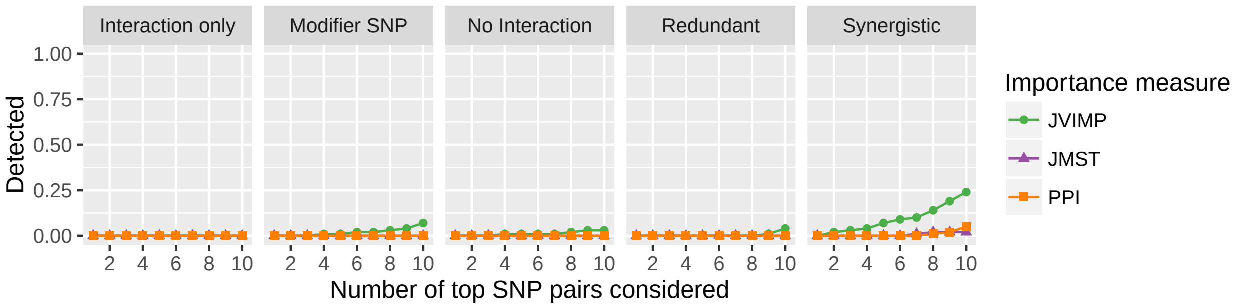

Figure S28: $\beta_I = 0.4, \beta_M = 0.8, MAF_I = 0.2, MAF_M = 0.2, mtry = 50$

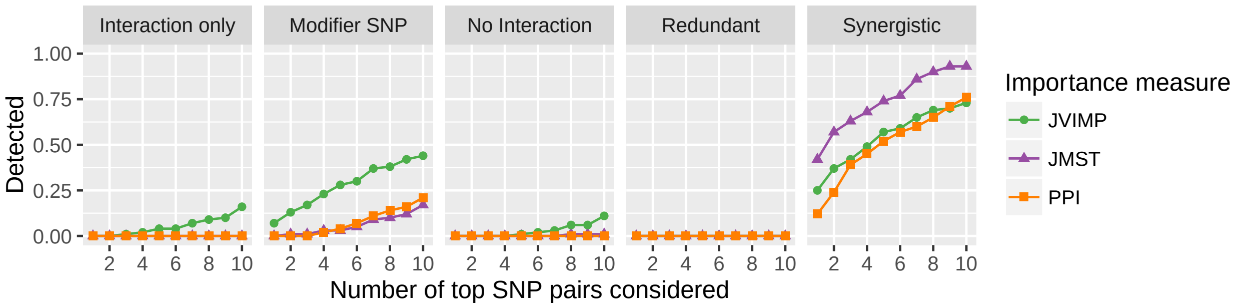

Figure S29: $\beta_I = 0.4, \beta_M = 0.8, MAF_I = 0.4, MAF_M = 0.2, mtry = 50$

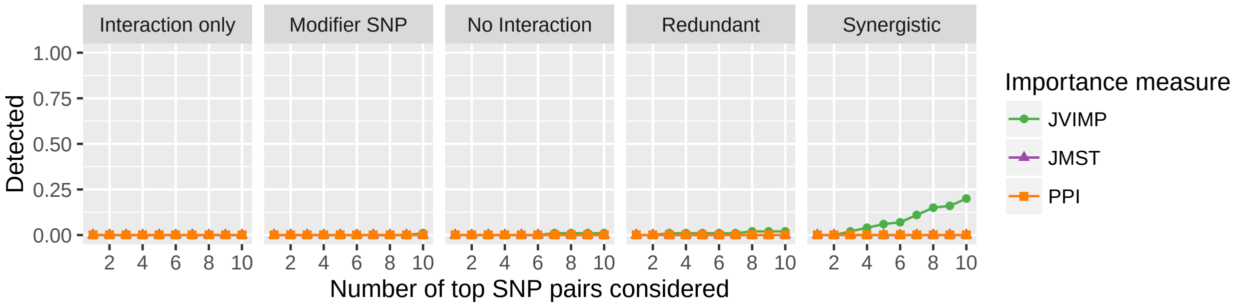

Figure S30: $\beta_I = 0.4, \beta_M = 0.8, MAF_I = 0.2, MAF_M = 0.4, mtry = 50$

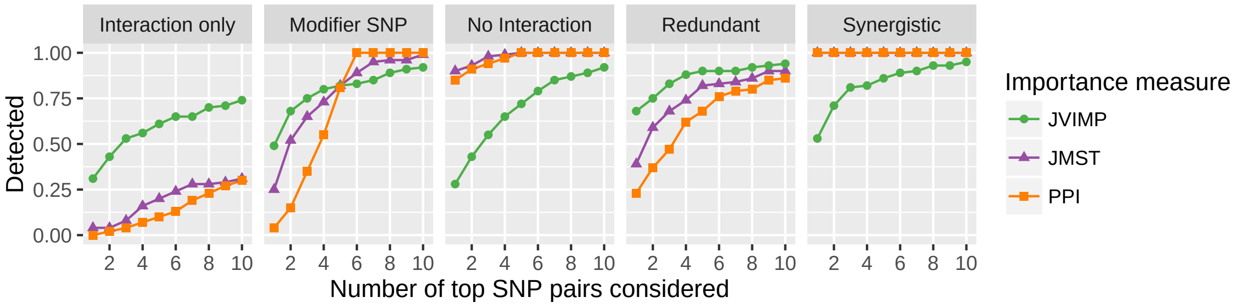

Figure S31: $\beta_I = 0.8, \beta_M = 0.4, MAF_I = 0.2, MAF_M = 0.2, mtry = 50$

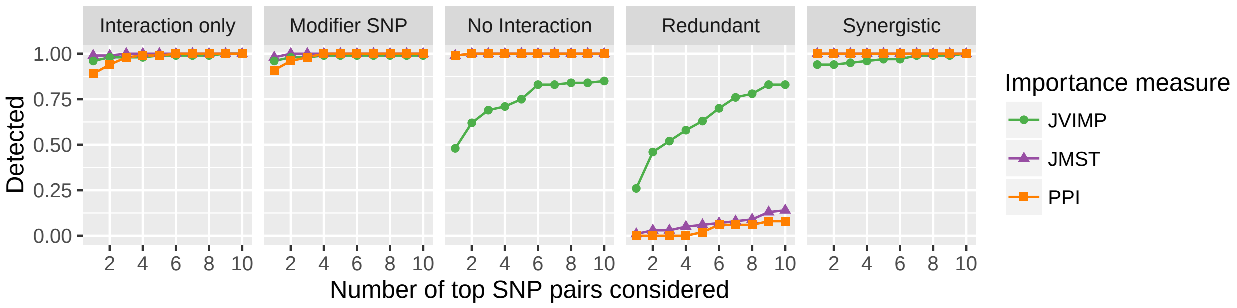

Figure S32: $\beta_I = 0.8, \beta_M = 0.4, MAF_I = 0.4, MAF_M = 0.2, mtry = 50$

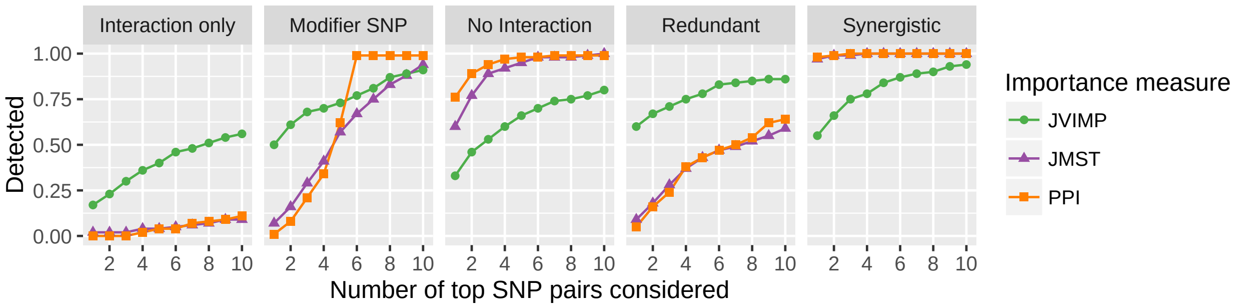

Figure S33: $\beta_I = 0.8, \beta_M = 0.4, MAF_I = 0.2, MAF_M = 0.4, mtry = 50$

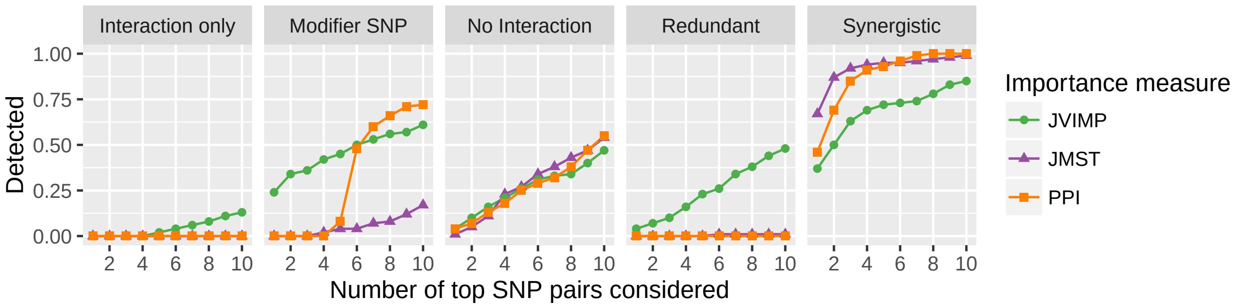

Figure S34: $\beta_I = 0.8, \beta_M = 0.8, MAF_I = 0.2, MAF_M = 0.2, mtry = 50$

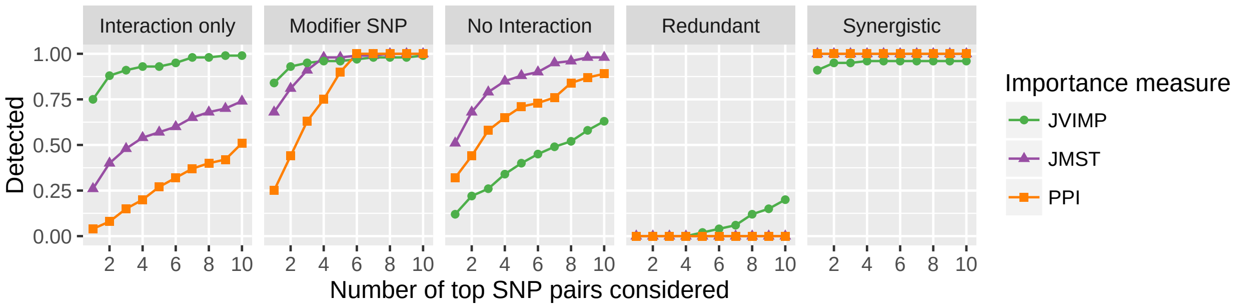

Figure S35: $\beta_I = 0.8, \beta_M = 0.8, MAF_I = 0.4, MAF_M = 0.2, mtry = 50$

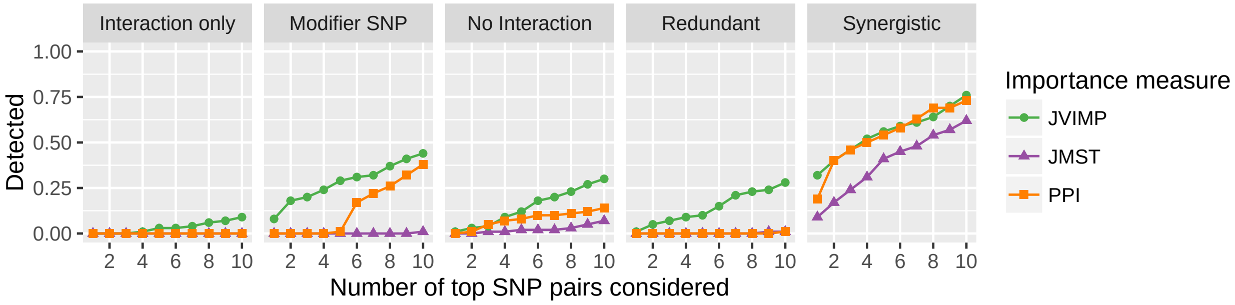

Figure S36: $\beta_I = 0.8, \beta_M = 0.8, MAF_I = 0.2, MAF_M = 0.4, mtry = 50$

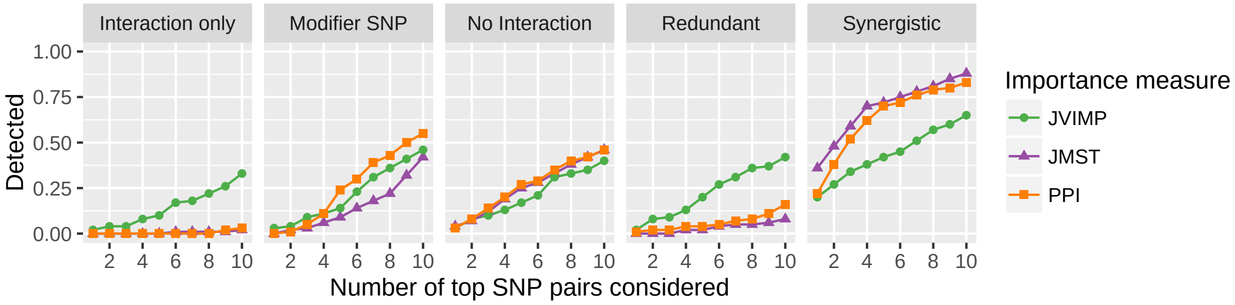

Figure S37: $\beta_I = 0.4, \beta_M = 0.4, MAF_I = 0.2, MAF_M = 0.2, mtry = 10$

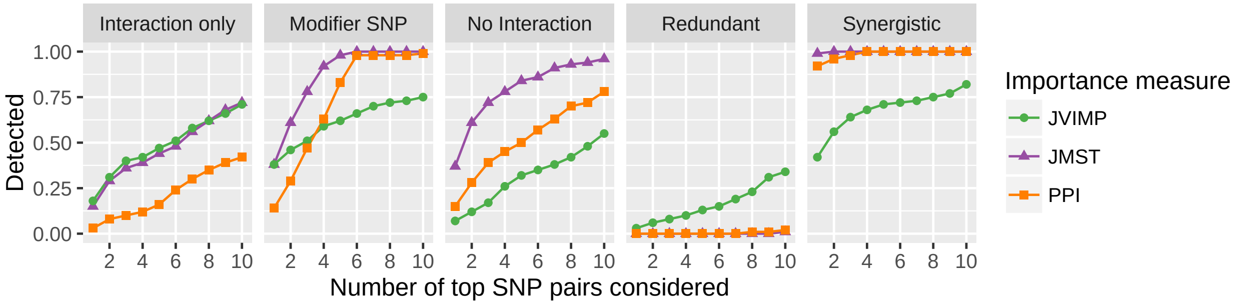

Figure S38: $\beta_I = 0.4, \beta_M = 0.4, MAF_I = 0.4, MAF_M = 0.2, mtry = 10$

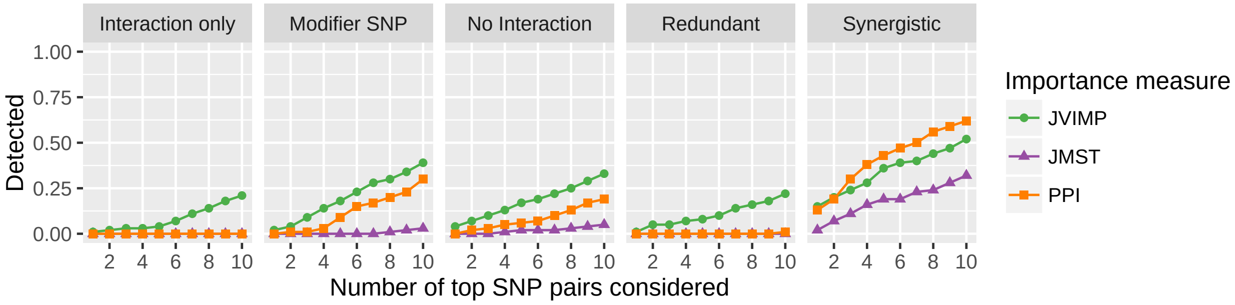

Figure S39: $\beta_I = 0.4, \beta_M = 0.4, MAF_I = 0.2, MAF_M = 0.4, mtry = 10$

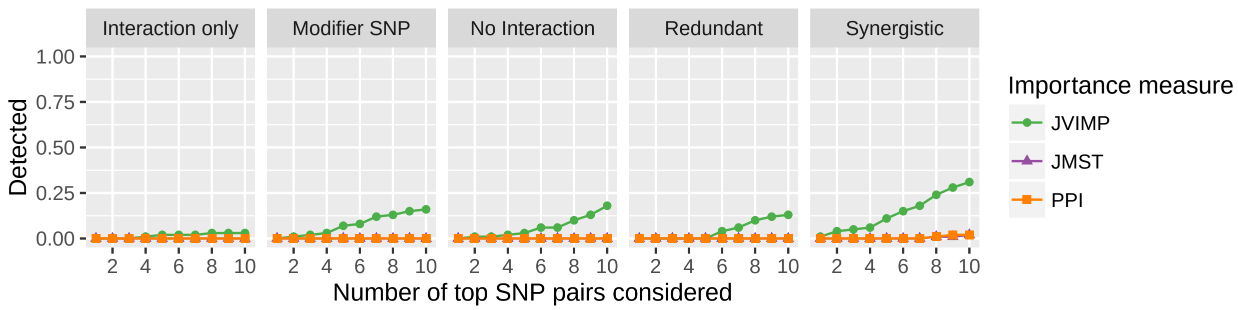

Figure S40: $\beta_I = 0.4, \beta_M = 0.8, MAF_I = 0.2, MAF_M = 0.2, mtry = 10$

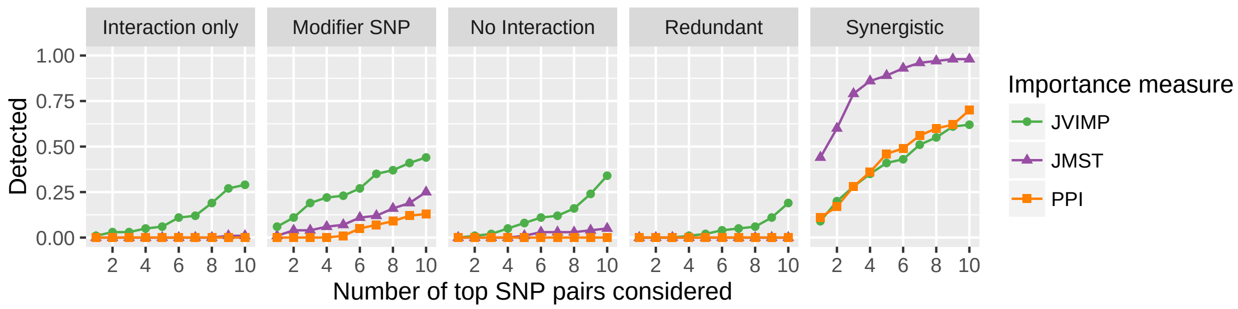

Figure S41: $\beta_I = 0.4, \beta_M = 0.8, MAF_I = 0.4, MAF_M = 0.2, mtry = 10$

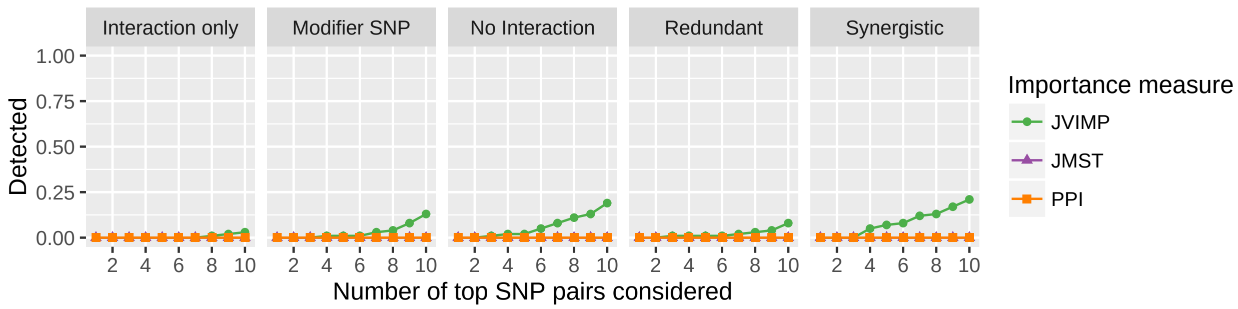

Figure S42: $\beta_I = 0.4, \beta_M = 0.8, MAF_I = 0.2, MAF_M = 0.4, mtry = 10$

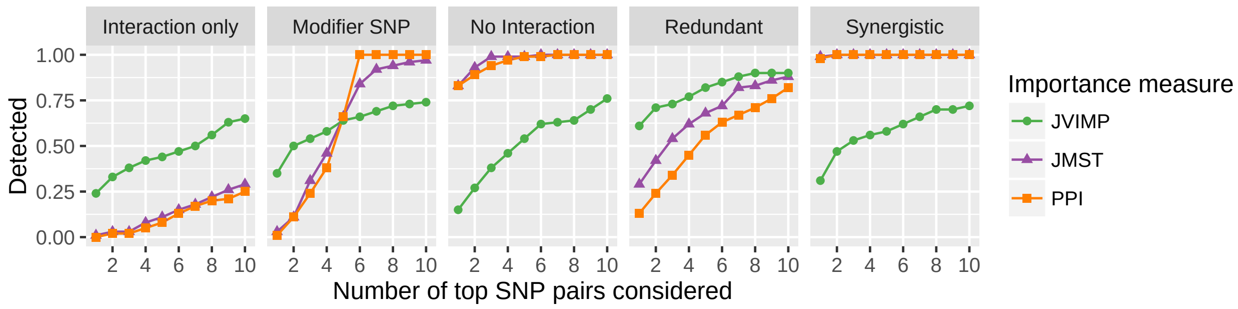

Figure S43: $\beta_I = 0.8, \beta_M = 0.4, MAF_I = 0.2, MAF_M = 0.2, mtry = 10$

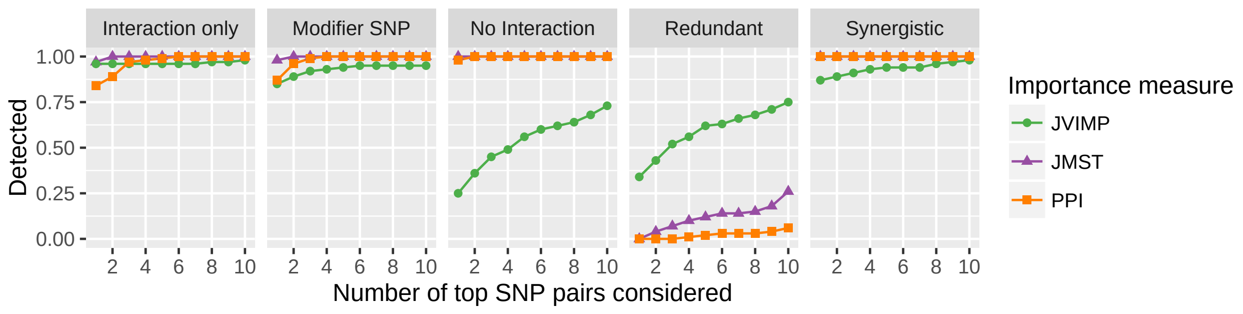

Figure S44: $\beta_I = 0.8, \beta_M = 0.4, MAF_I = 0.4, MAF_M = 0.2, mtry = 10$

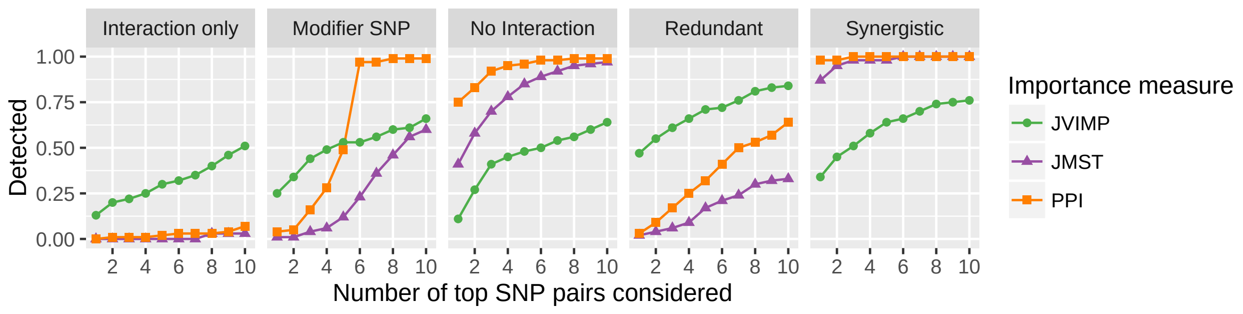

Figure S45: $\beta_I = 0.8, \beta_M = 0.4, MAF_I = 0.2, MAF_M = 0.4, mtry = 10$

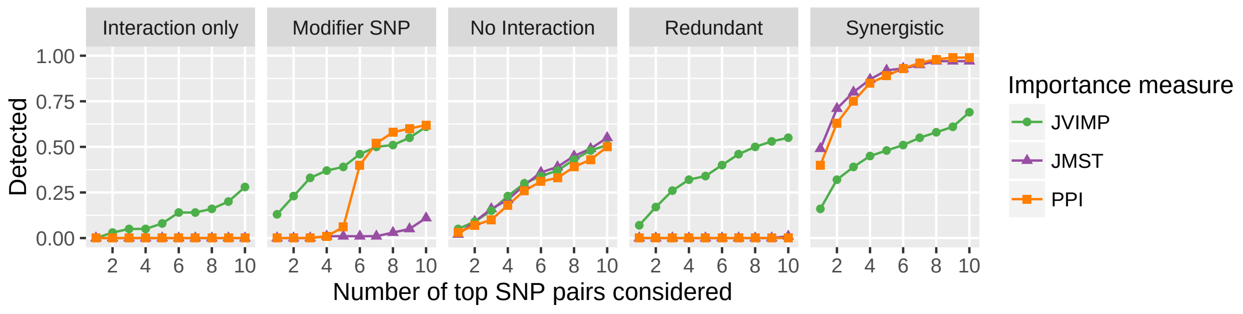

Figure S46: $\beta_I = 0.8, \beta_M = 0.8, MAF_I = 0.2, MAF_M = 0.2, mtry = 10$

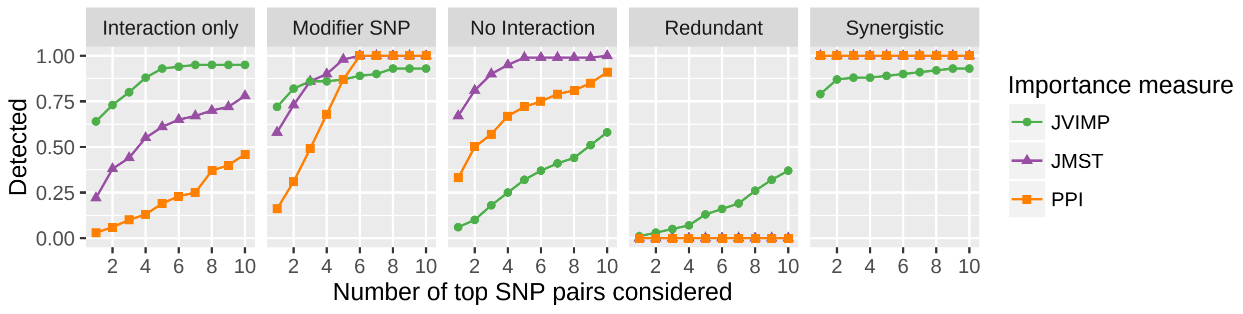

Figure S47: $\beta_I = 0.8, \beta_M = 0.8, MAF_I = 0.4, MAF_M = 0.2, mtry = 10$

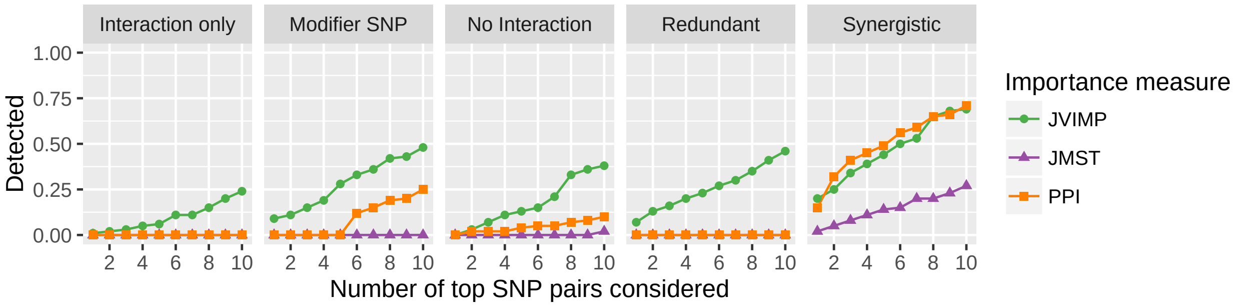

Figure S48: $\beta_I = 0.8, \beta_M = 0.8, MAF_I = 0.2, MAF_M = 0.4, mtry = 10$

Supplement for: *Do little interactions get lost in dark random forests?*

Part 4: Ranks of marginal-only SNPs, single variable importance measures

Marvin N. Wright, Andreas Ziegler, Inke R. König

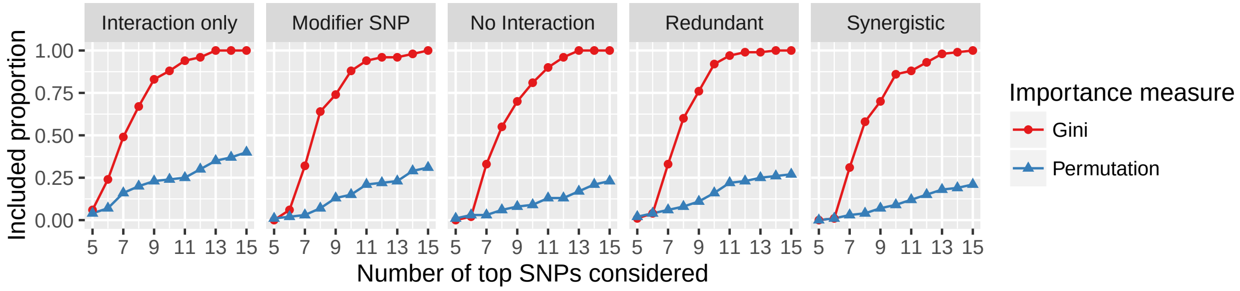

Figure S49: $\beta_I = 0.4, \beta_M = 0.4, MAF_I = 0.2, MAF_M = 0.2, mtry = 50$

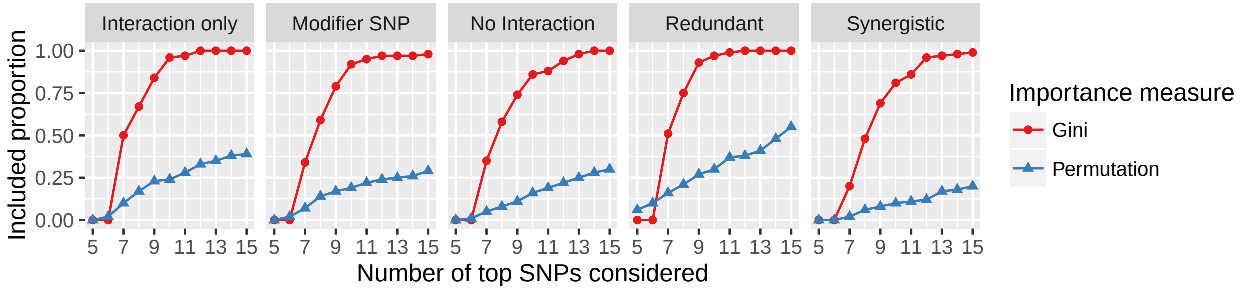

Figure S50: $\beta_I = 0.4, \beta_M = 0.4, MAF_I = 0.4, MAF_M = 0.2, mtry = 50$

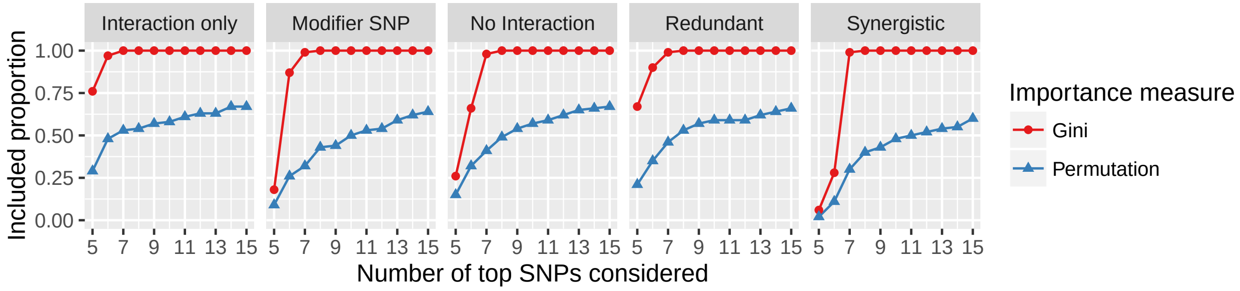

Figure S51: $\beta_I = 0.4, \beta_M = 0.4, MAF_I = 0.2, MAF_M = 0.4, mtry = 50$

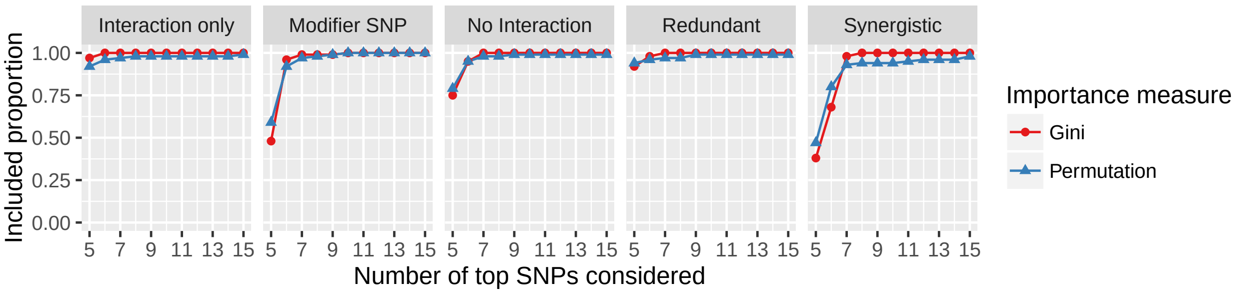

Figure S52: $\beta_I = 0.4, \beta_M = 0.8, MAF_I = 0.2, MAF_M = 0.2, mtry = 50$

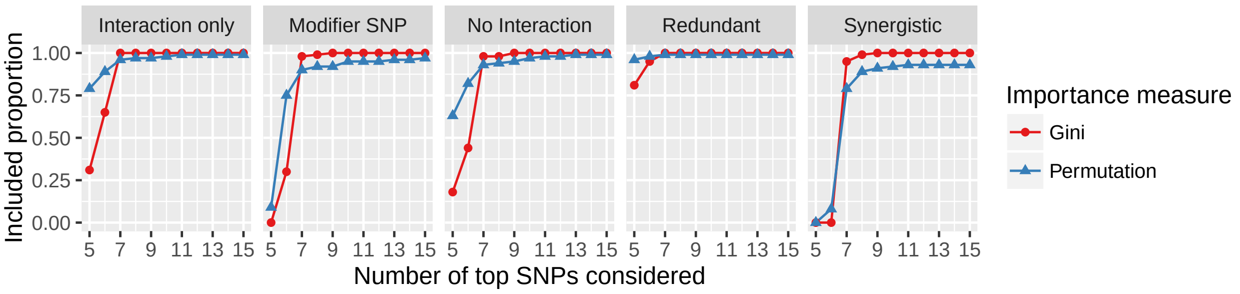

Figure S53: $\beta_I = 0.4, \beta_M = 0.8, MAF_I = 0.4, MAF_M = 0.2, mtry = 50$

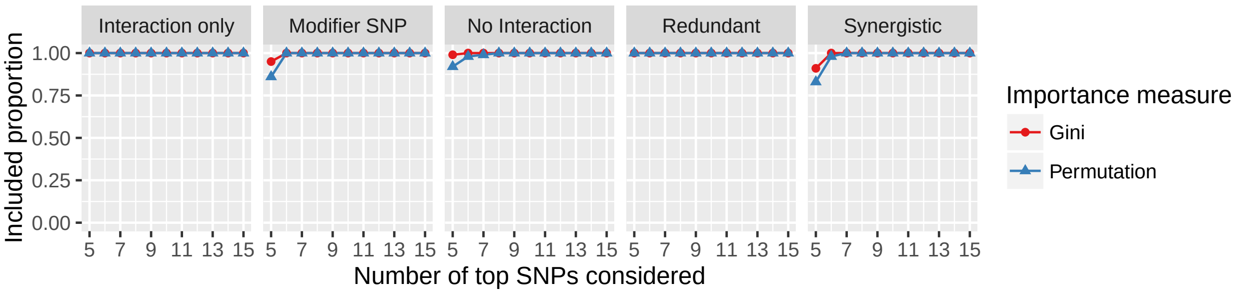

Figure S54: $\beta_I = 0.4, \beta_M = 0.8, MAF_I = 0.2, MAF_M = 0.4, mtry = 50$

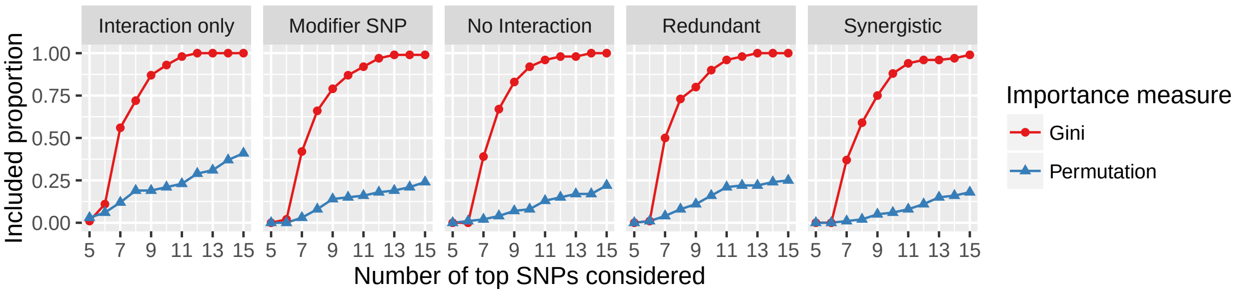

Figure S55: $\beta_I = 0.8, \beta_M = 0.4, MAF_I = 0.2, MAF_M = 0.2, mtry = 50$

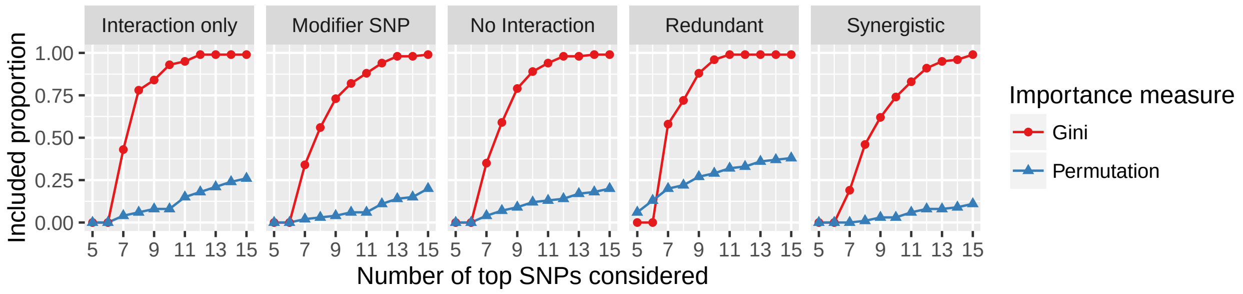

Figure S56: $\beta_I = 0.8, \beta_M = 0.4, MAF_I = 0.4, MAF_M = 0.2, mtry = 50$

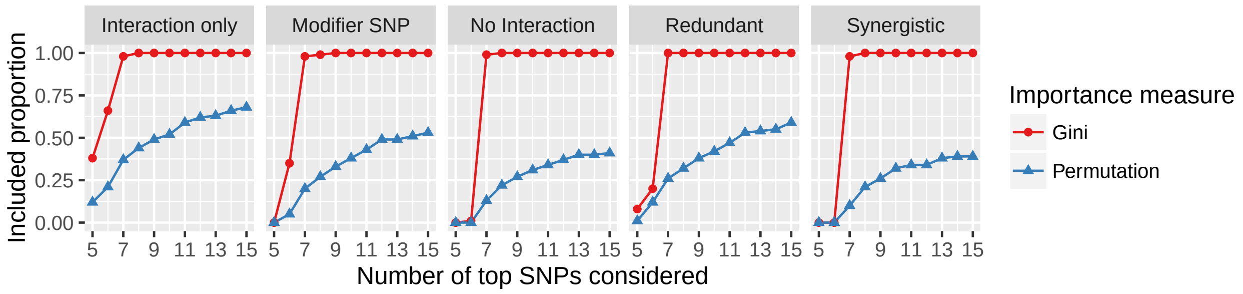

Figure S57: $\beta_I = 0.8, \beta_M = 0.4, MAF_I = 0.2, MAF_M = 0.4, mtry = 50$

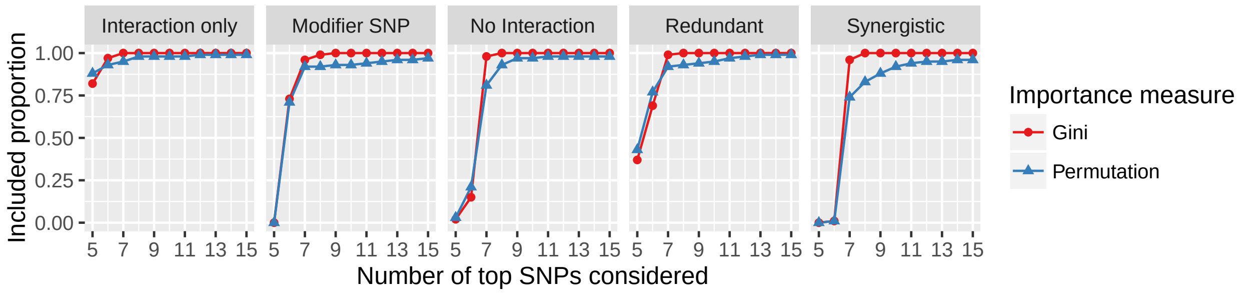

Figure S58: $\beta_I = 0.8, \beta_M = 0.8, MAF_I = 0.2, MAF_M = 0.2, mtry = 50$

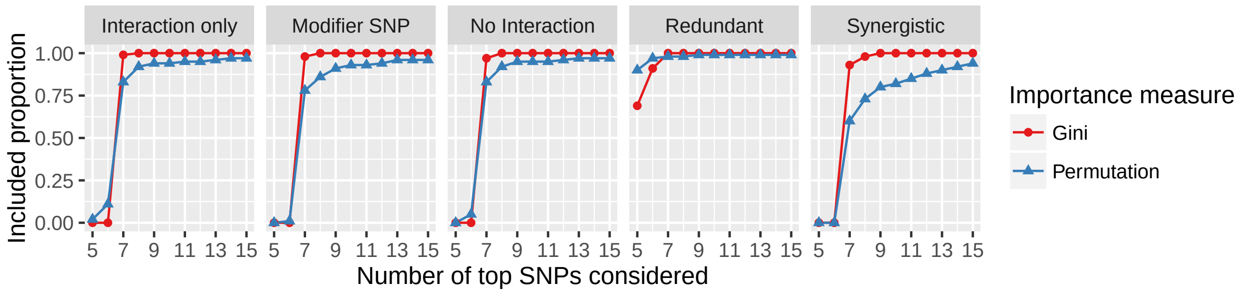

Figure S59: $\beta_I = 0.8, \beta_M = 0.8, MAF_I = 0.4, MAF_M = 0.2, mtry = 50$

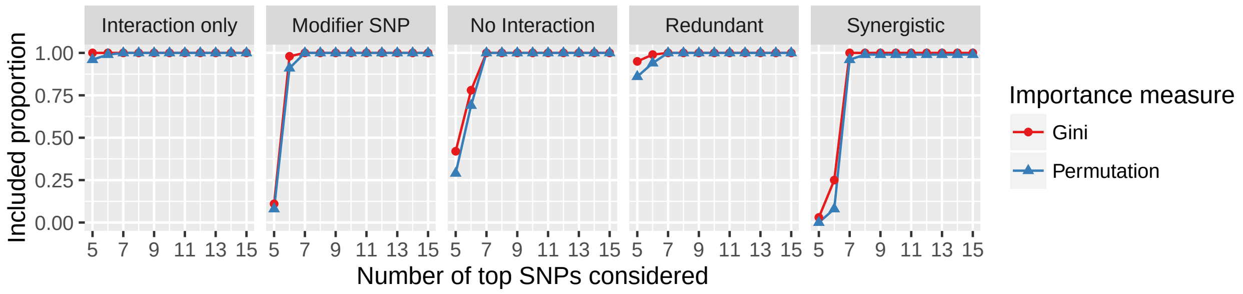

Figure S60: $\beta_I = 0.8, \beta_M = 0.8, MAF_I = 0.2, MAF_M = 0.4, mtry = 50$

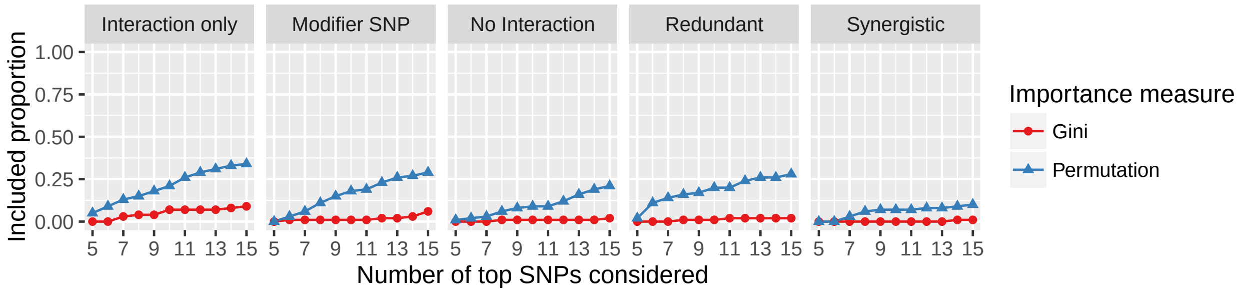

Figure S61: $\beta_I = 0.4, \beta_M = 0.4, MAF_I = 0.2, MAF_M = 0.2, mtry = 10$

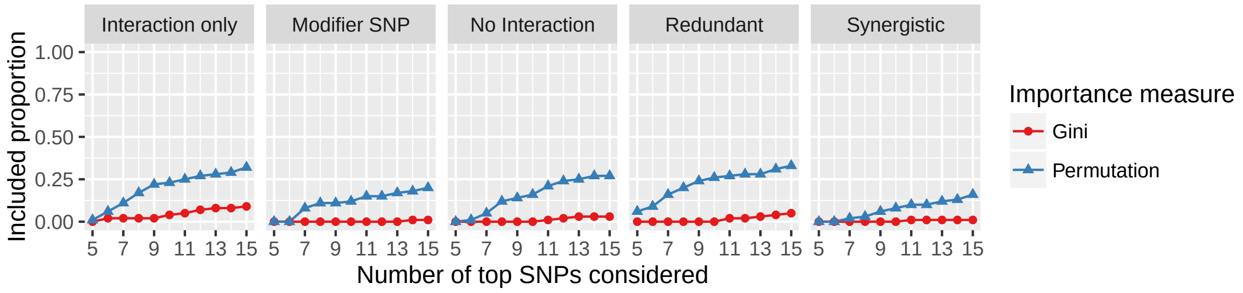

Figure S62: $\beta_I = 0.4, \beta_M = 0.4, MAF_I = 0.4, MAF_M = 0.2, mtry = 10$

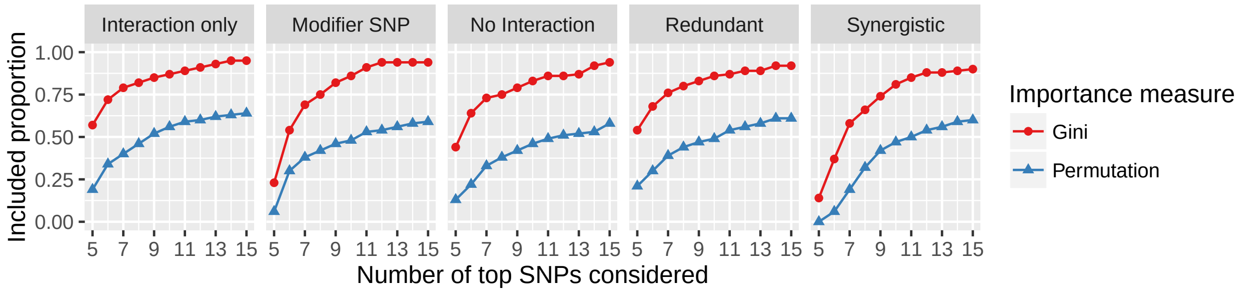

Figure S63: $\beta_I = 0.4, \beta_M = 0.4, MAF_I = 0.2, MAF_M = 0.4, mtry = 10$

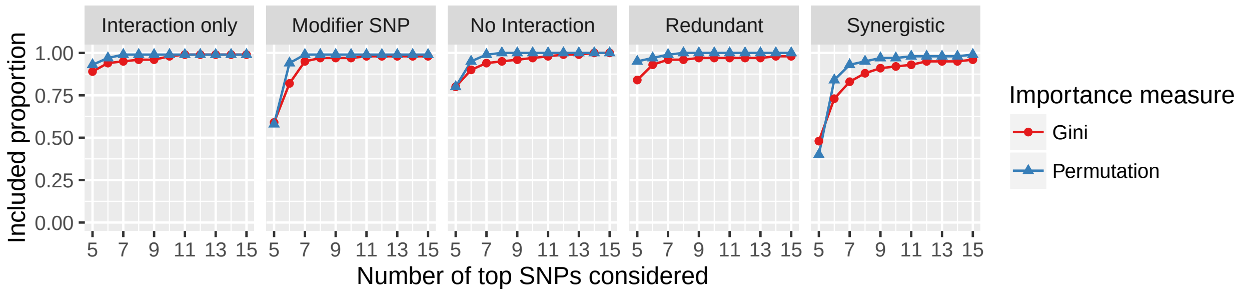

Figure S64: $\beta_I = 0.4, \beta_M = 0.8, MAF_I = 0.2, MAF_M = 0.2, mtry = 10$

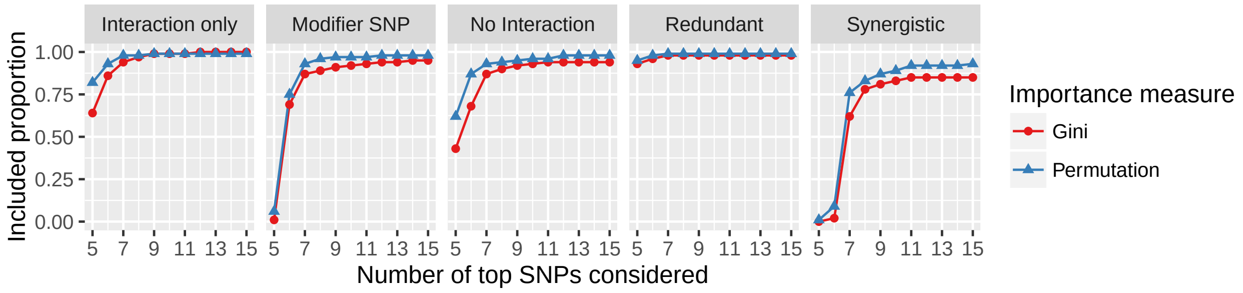

Figure S65: $\beta_I = 0.4, \beta_M = 0.8, MAF_I = 0.4, MAF_M = 0.2, mtry = 10$

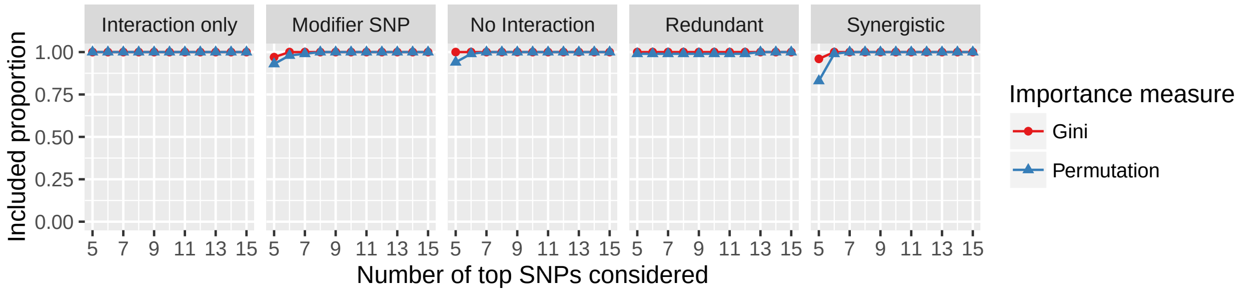

Figure S66: $\beta_I = 0.4, \beta_M = 0.8, MAF_I = 0.2, MAF_M = 0.4, mtry = 10$

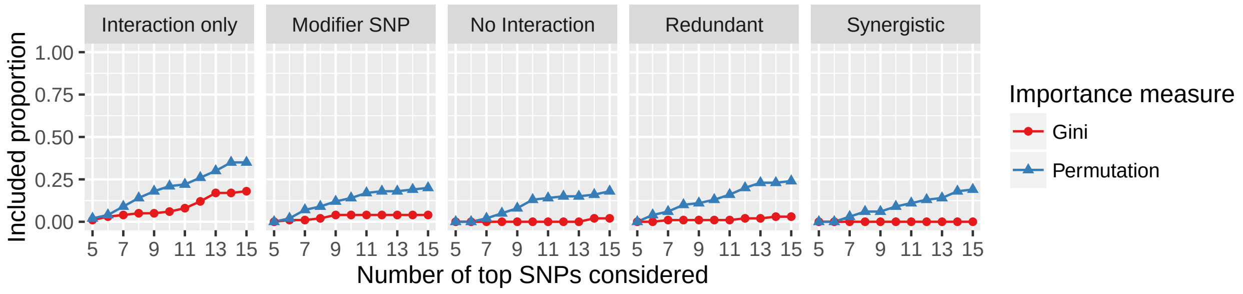

Figure S67: $\beta_I = 0.8, \beta_M = 0.4, MAF_I = 0.2, MAF_M = 0.2, mtry = 10$

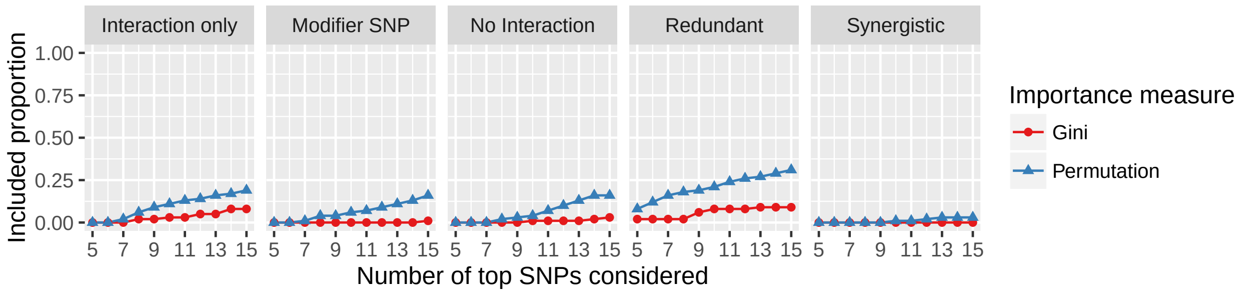

Figure S68: $\beta_I = 0.8, \beta_M = 0.4, MAF_I = 0.4, MAF_M = 0.2, mtry = 10$

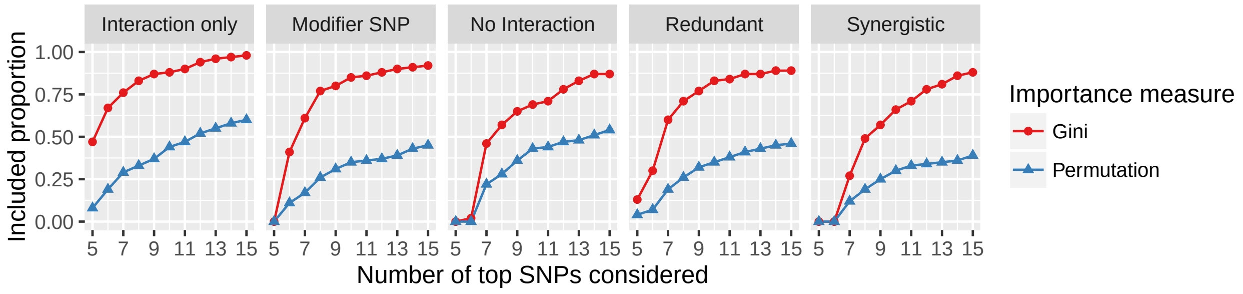

Figure S69: $\beta_I = 0.8, \beta_M = 0.4, MAF_I = 0.2, MAF_M = 0.4, mtry = 10$

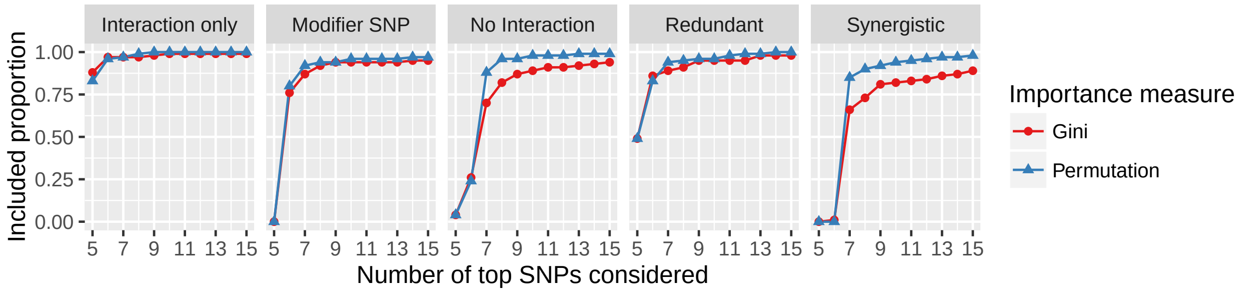

Figure S70: $\beta_I = 0.8, \beta_M = 0.8, MAF_I = 0.2, MAF_M = 0.2, mtry = 10$

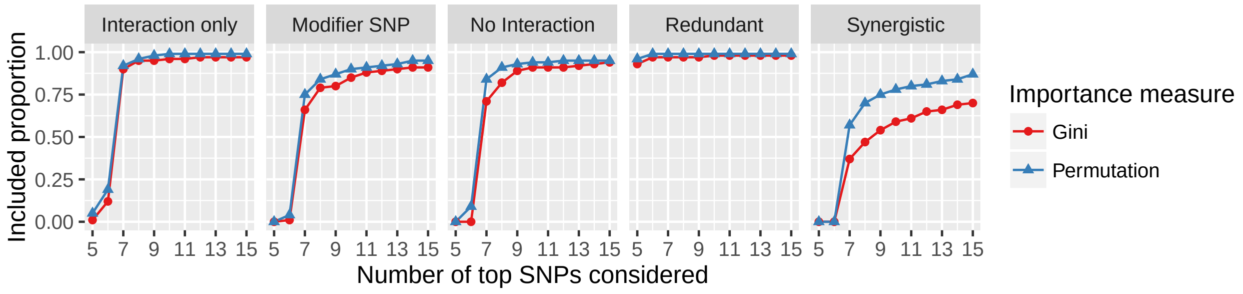

Figure S71: $\beta_I = 0.8, \beta_M = 0.8, MAF_I = 0.4, MAF_M = 0.2, mtry = 10$

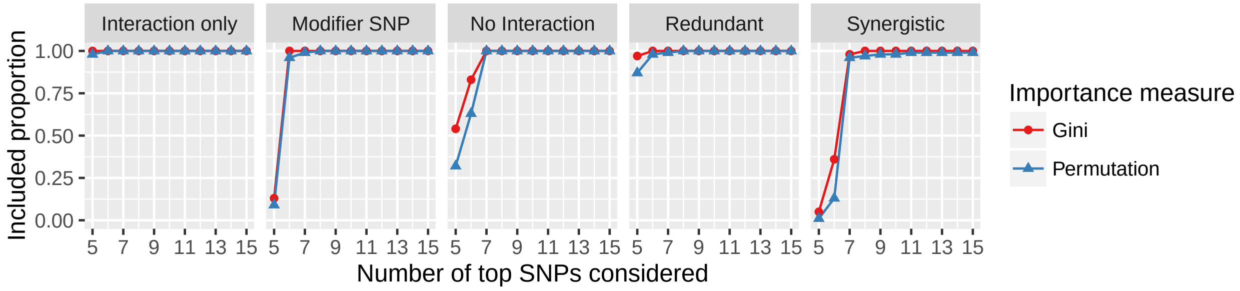

Figure S72: $\beta_I = 0.8, \beta_M = 0.8, MAF_I = 0.2, MAF_M = 0.4, mtry = 10$

Supplement for: *Do little interactions get lost in dark random forests?*

Part 5: Ranks of marginal-only SNPs, pairwise variable importance measures

Marvin N. Wright, Andreas Ziegler, Inke R. König

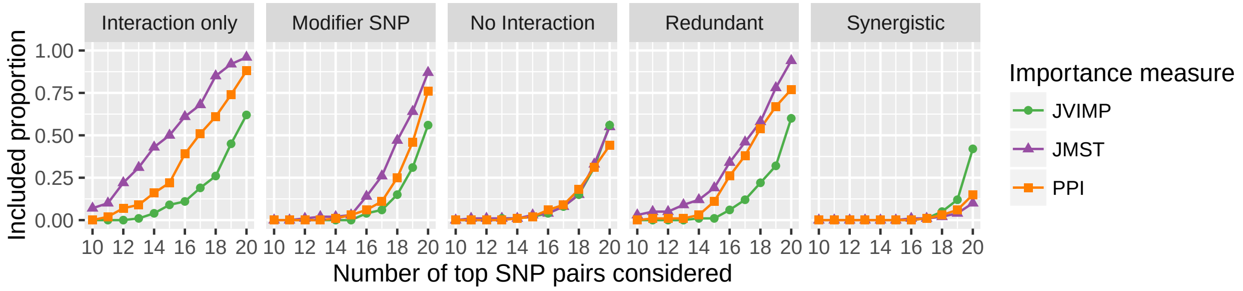

Figure S73: $\beta_I = 0.4, \beta_M = 0.4, MAF_I = 0.2, MAF_M = 0.2, mtry = 50$

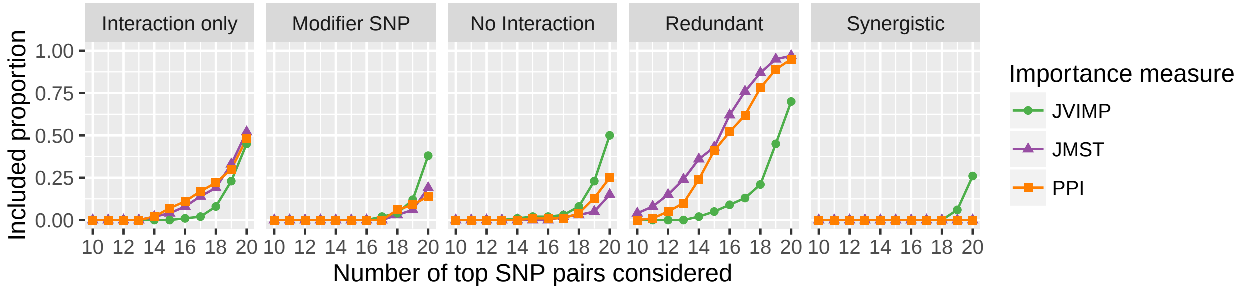

Figure S74: $\beta_I = 0.4, \beta_M = 0.4, MAF_I = 0.4, MAF_M = 0.2, mtry = 50$

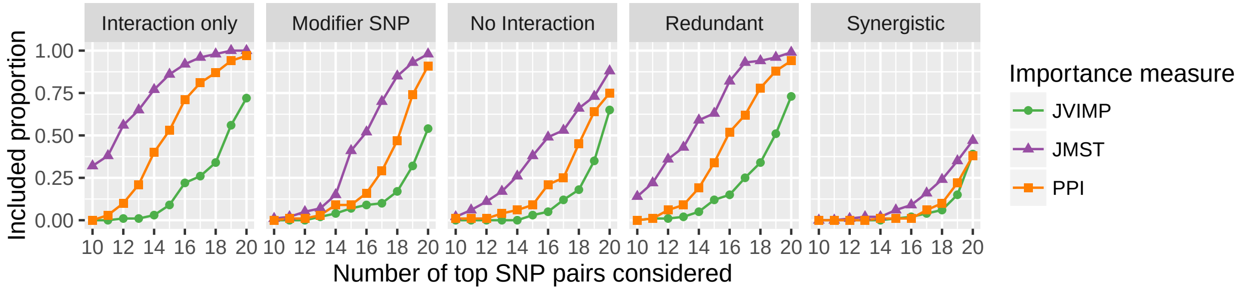

Figure S75: $\beta_I = 0.4, \beta_M = 0.4, MAF_I = 0.2, MAF_M = 0.4, mtry = 50$

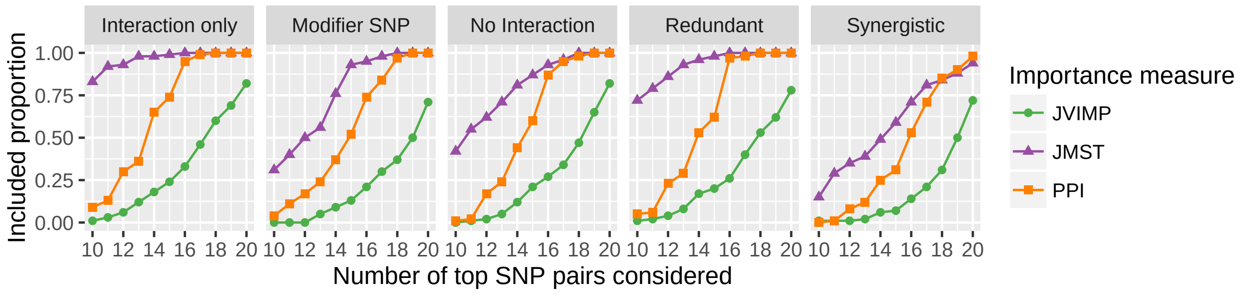

Figure S76: $\beta_I = 0.4, \beta_M = 0.8, MAF_I = 0.2, MAF_M = 0.2, mtry = 50$

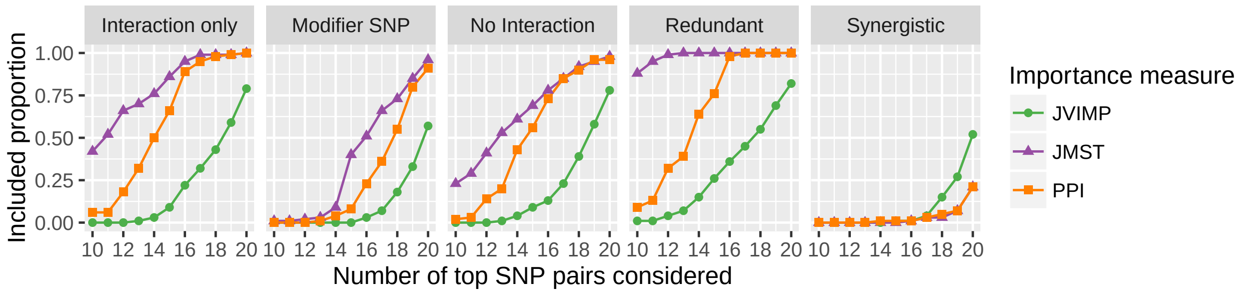

Figure S77: $\beta_I = 0.4, \beta_M = 0.8, MAF_I = 0.4, MAF_M = 0.2, mtry = 50$

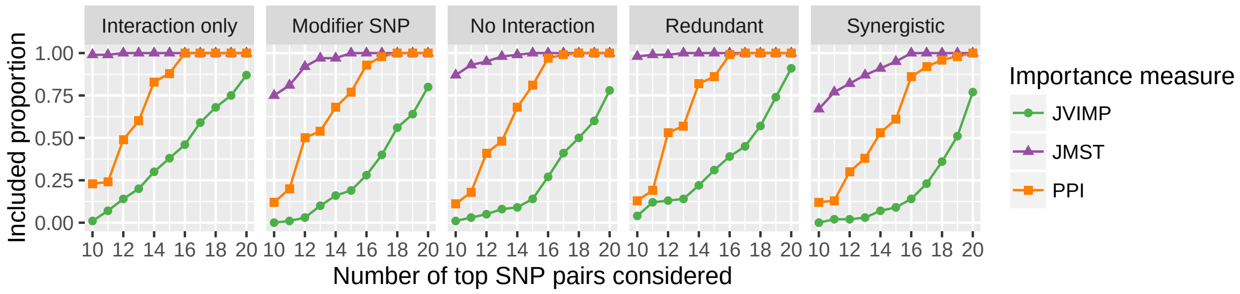

Figure S78: $\beta_I = 0.4, \beta_M = 0.8, MAF_I = 0.2, MAF_M = 0.4, mtry = 50$

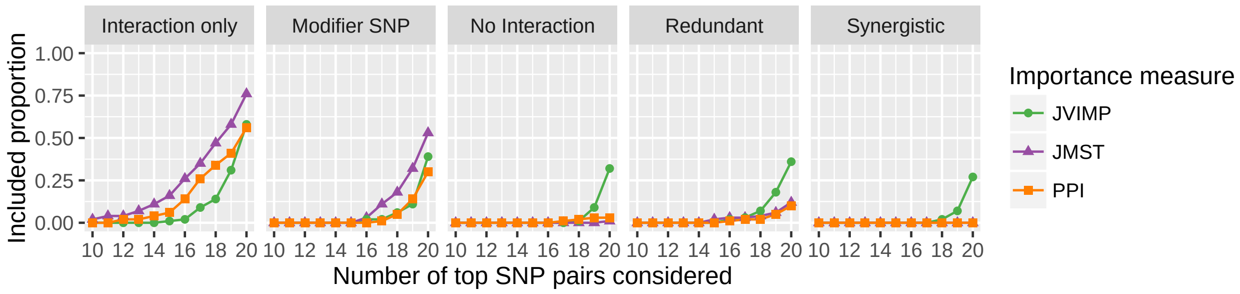

Figure S79: $\beta_I = 0.8, \beta_M = 0.4, MAF_I = 0.2, MAF_M = 0.2, mtry = 50$

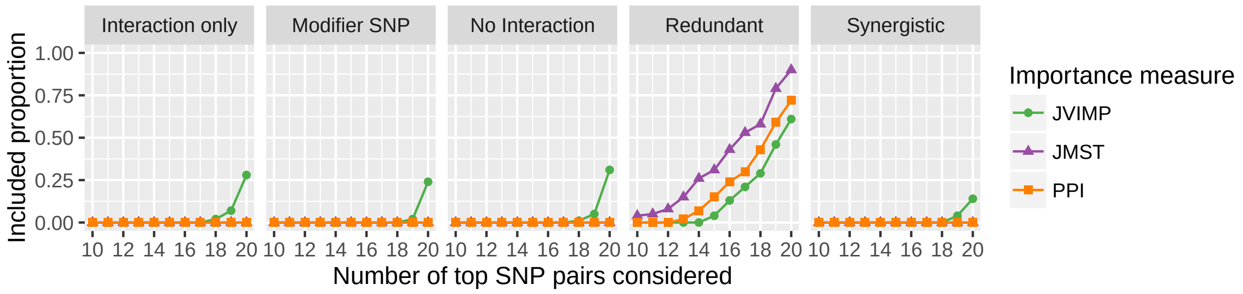

Figure S80: $\beta_I = 0.8, \beta_M = 0.4, MAF_I = 0.4, MAF_M = 0.2, mtry = 50$

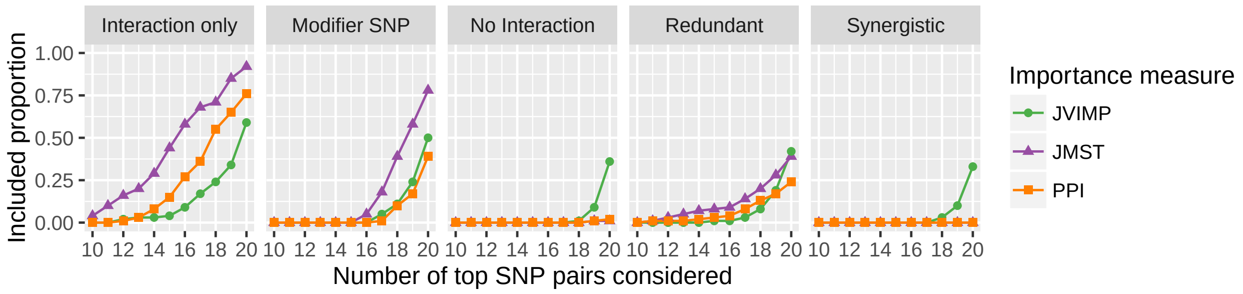

Figure S81: $\beta_I = 0.8, \beta_M = 0.4, MAF_I = 0.2, MAF_M = 0.4, mtry = 50$

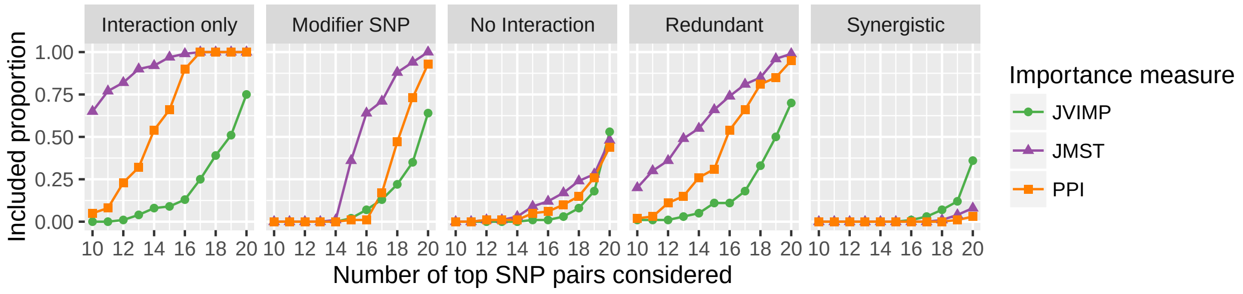

Figure S82: $\beta_I = 0.8, \beta_M = 0.8, MAF_I = 0.2, MAF_M = 0.2, mtry = 50$

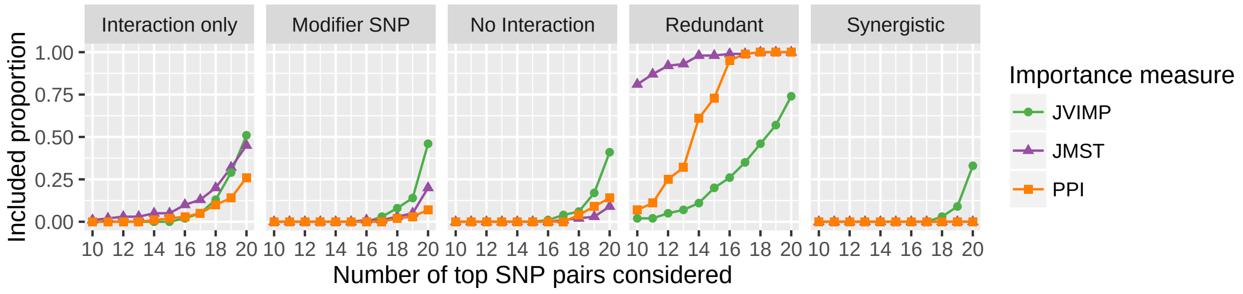

Figure S83: $\beta_I = 0.8, \beta_M = 0.8, MAF_I = 0.4, MAF_M = 0.2, mtry = 50$

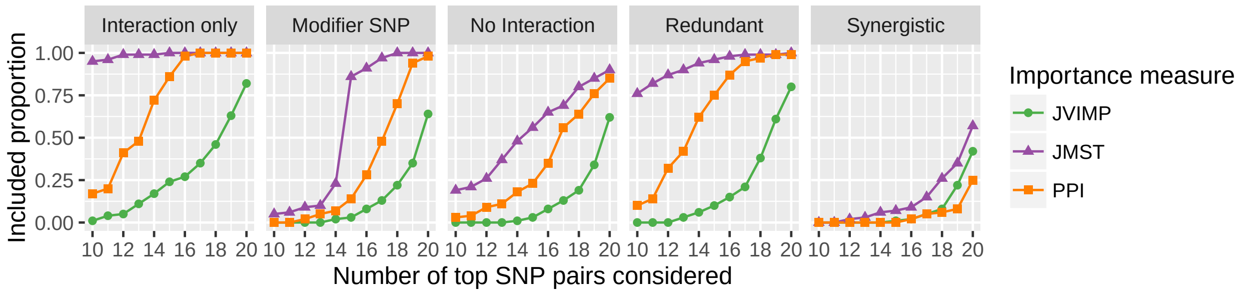

Figure S84: $\beta_I = 0.8, \beta_M = 0.8, MAF_I = 0.2, MAF_M = 0.4, mtry = 50$

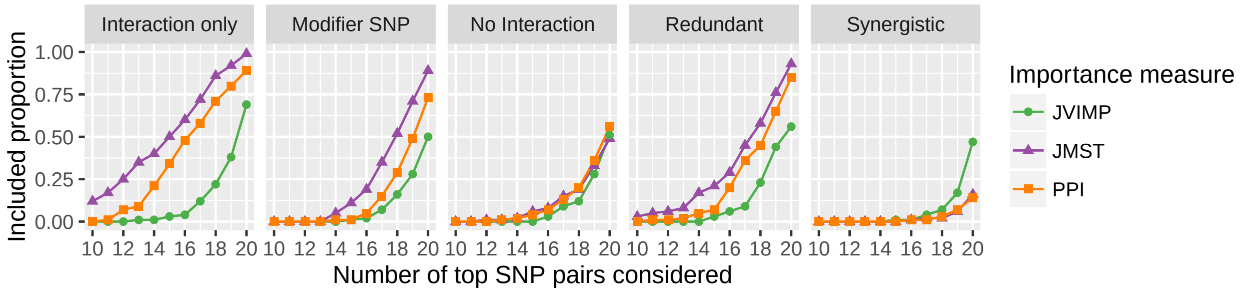

Figure S85: $\beta_I = 0.4, \beta_M = 0.4, MAF_I = 0.2, MAF_M = 0.2, mtry = 10$

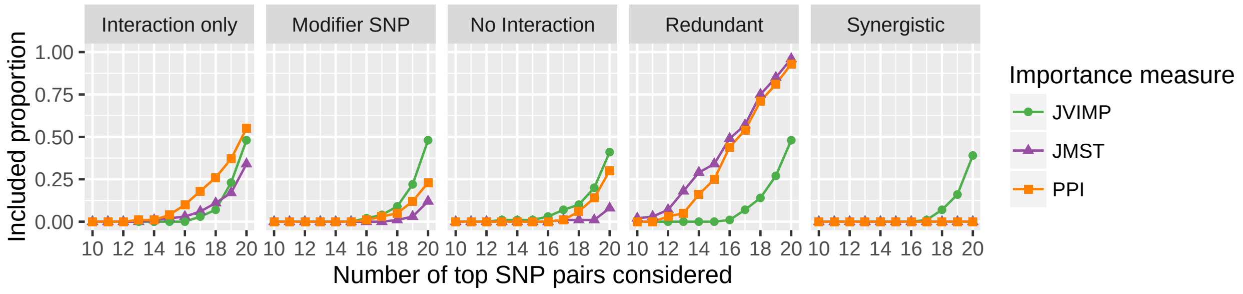

Figure S86: $\beta_I = 0.4, \beta_M = 0.4, MAF_I = 0.4, MAF_M = 0.2, mtry = 10$

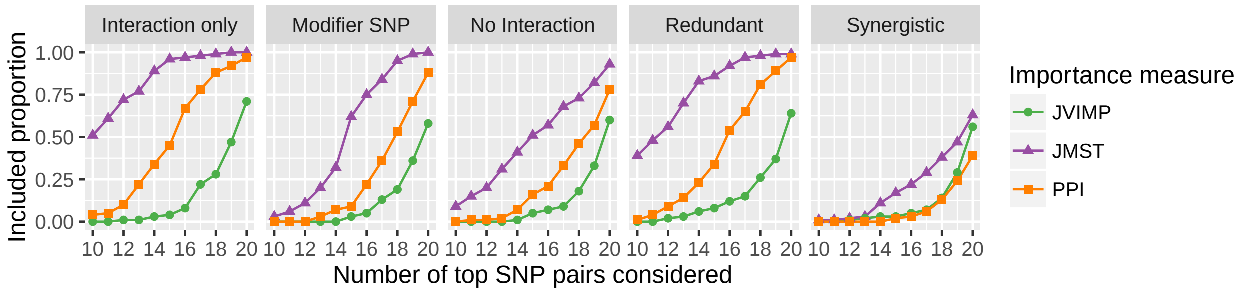

Figure S87: $\beta_I = 0.4, \beta_M = 0.4, MAF_I = 0.2, MAF_M = 0.4, mtry = 10$

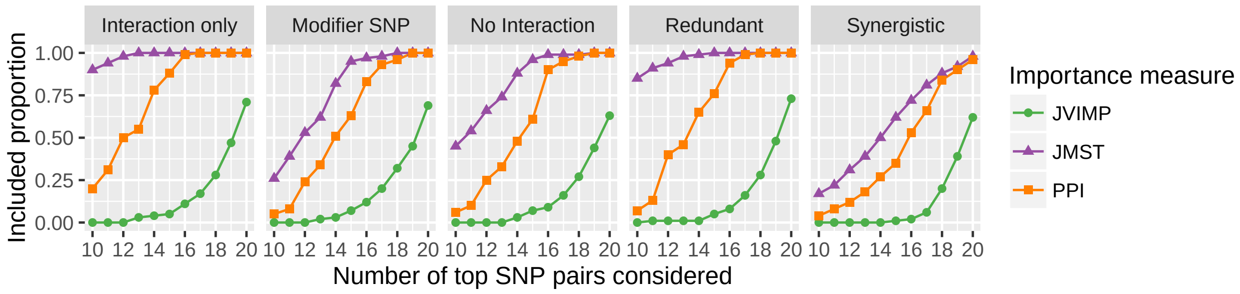

Figure S88: $\beta_I = 0.4, \beta_M = 0.8, MAF_I = 0.2, MAF_M = 0.2, mtry = 10$

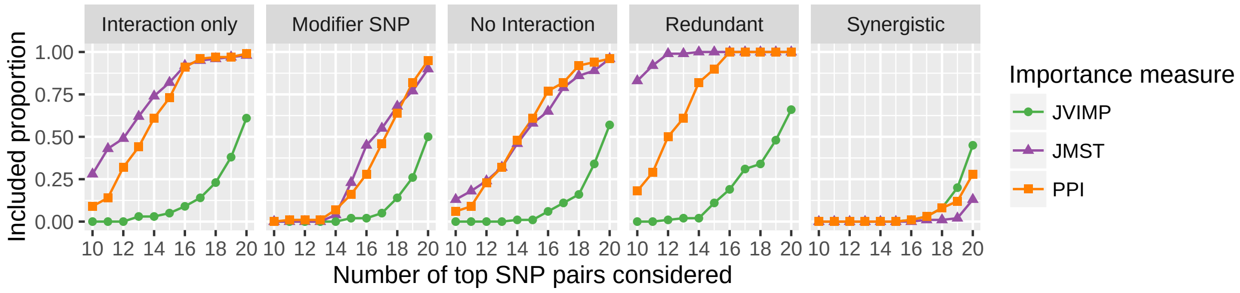

Figure S89: $\beta_I = 0.4, \beta_M = 0.8, MAF_I = 0.4, MAF_M = 0.2, mtry = 10$

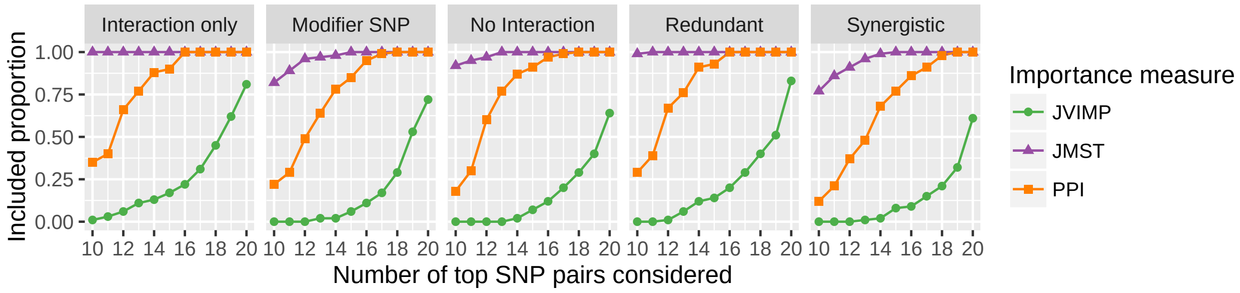

Figure S90: $\beta_I = 0.4, \beta_M = 0.8, MAF_I = 0.2, MAF_M = 0.4, mtry = 10$

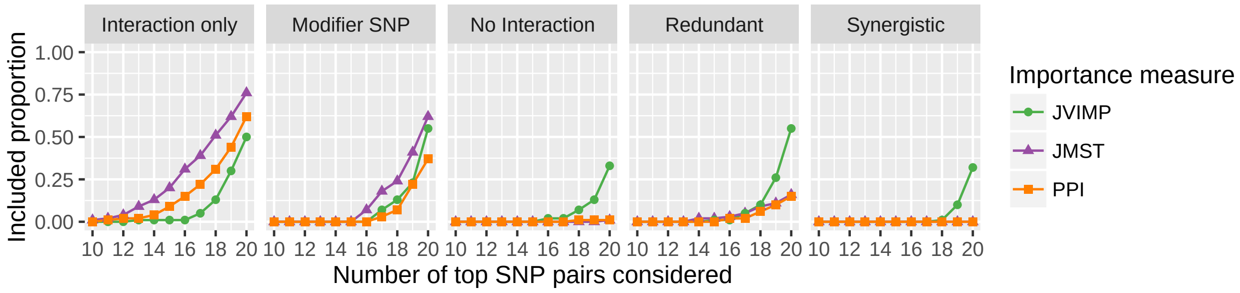

Figure S91: $\beta_I = 0.8, \beta_M = 0.4, MAF_I = 0.2, MAF_M = 0.2, mtry = 10$

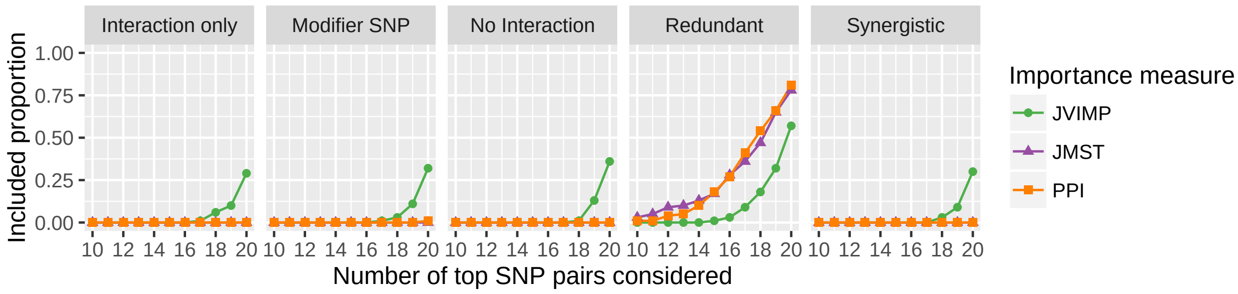

Figure S92: $\beta_I = 0.8, \beta_M = 0.4, MAF_I = 0.4, MAF_M = 0.2, mtry = 10$

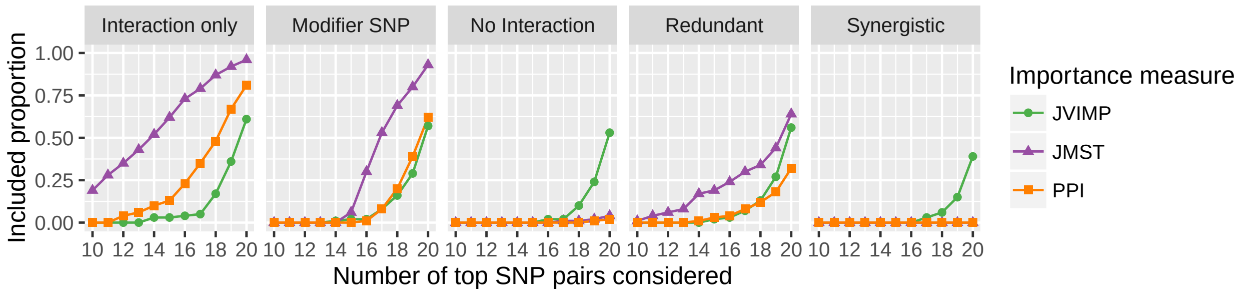

Figure S93: $\beta_I = 0.8, \beta_M = 0.4, MAF_I = 0.2, MAF_M = 0.4, mtry = 10$

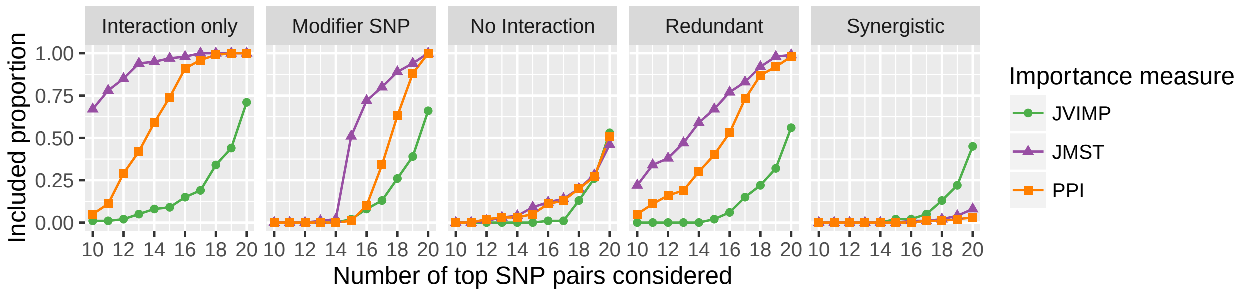

Figure S94: $\beta_I = 0.8, \beta_M = 0.8, MAF_I = 0.2, MAF_M = 0.2, mtry = 10$

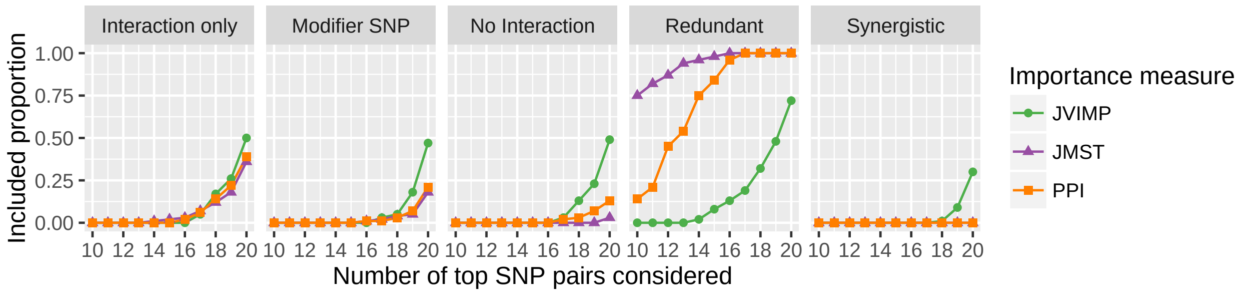

Figure S95: $\beta_I = 0.8, \beta_M = 0.8, MAF_I = 0.4, MAF_M = 0.2, mtry = 10$

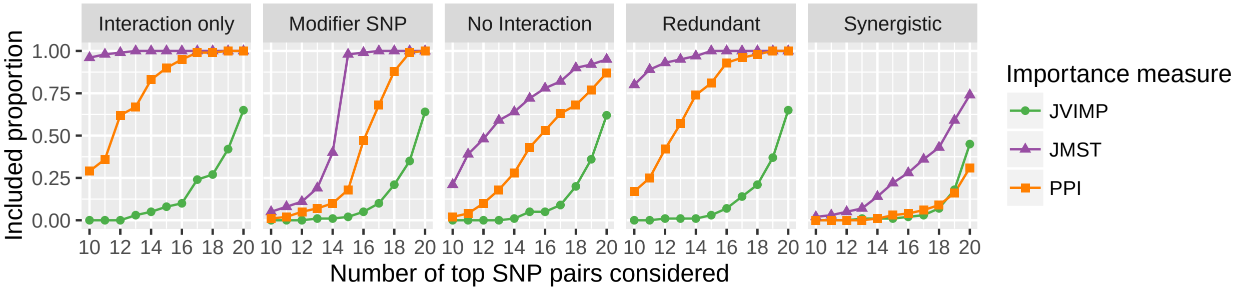

Figure S96: $\beta_I = 0.8, \beta_M = 0.8, MAF_I = 0.2, MAF_M = 0.4, mtry = 10$

Supplement for: *Do little interactions get lost in dark random forests?*

Part 6: Additional scenarios, single variable importance measures

Marvin N. Wright, Andreas Ziegler, Inke R. König

1 Fewer marginal-only SNPs

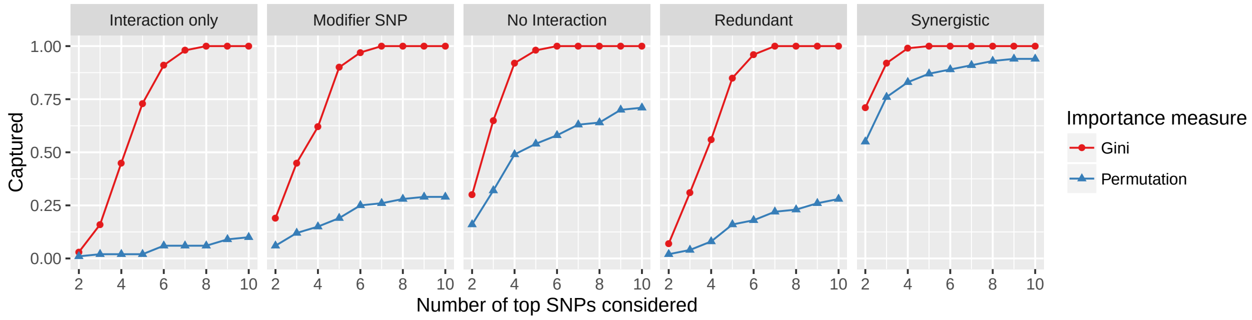

Figure S97: $\beta_I = 0.4, \beta_M = 0.4, MAF_I = 0.2, MAF_M = 0.2, mtry = 50$. 2 marginal-only SNPs.

2 More SNPs than samples

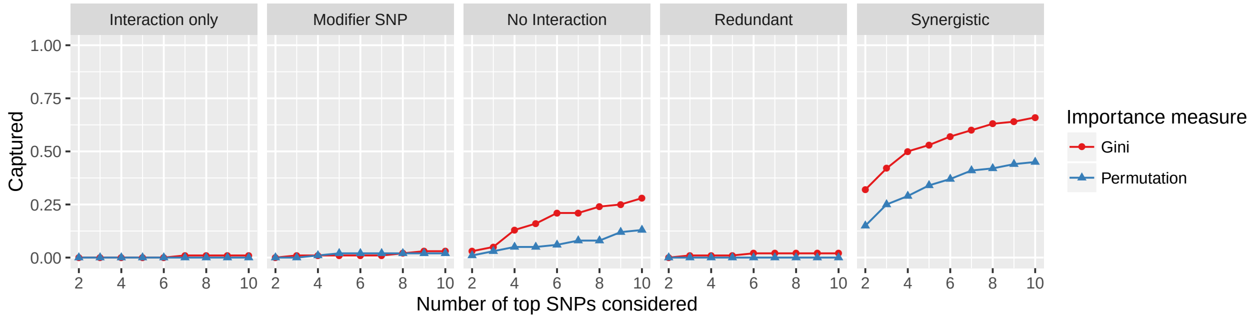

Figure S98: $\beta_I = 0.4, \beta_M = 0.4, MAF_I = 0.2, MAF_M = 0.2, mtry = 50, 2500$ SNPs.

3 Correlated SNPs

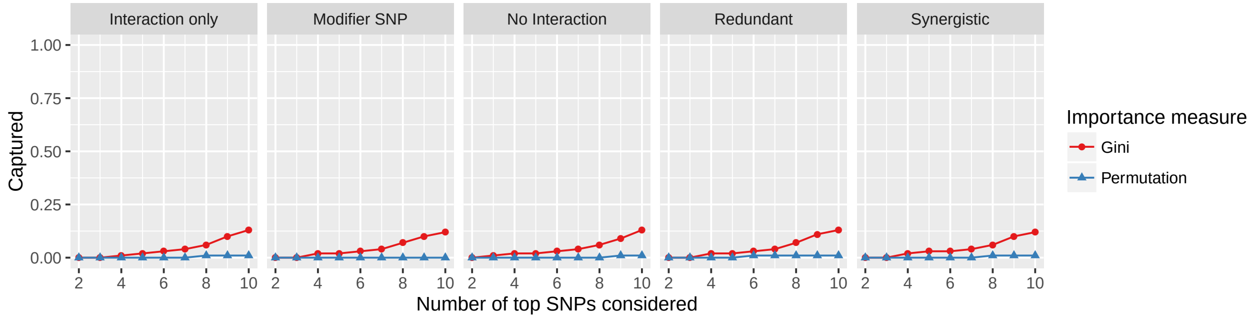

Figure S99: $\beta_I = 0, \beta_M = 0, mtry = 50$. Linkage disequilibrium.

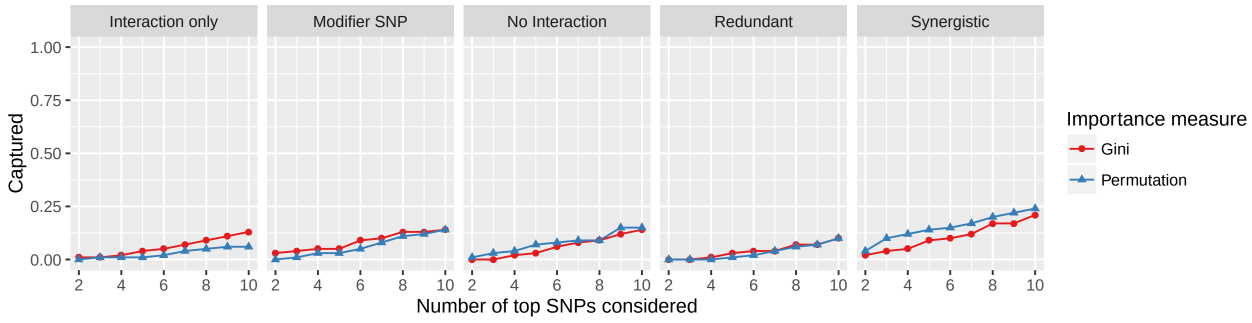

Figure S100: $\beta_I = 0.4, \beta_M = 0.4, mtry = 50$. Linkage disequilibrium.

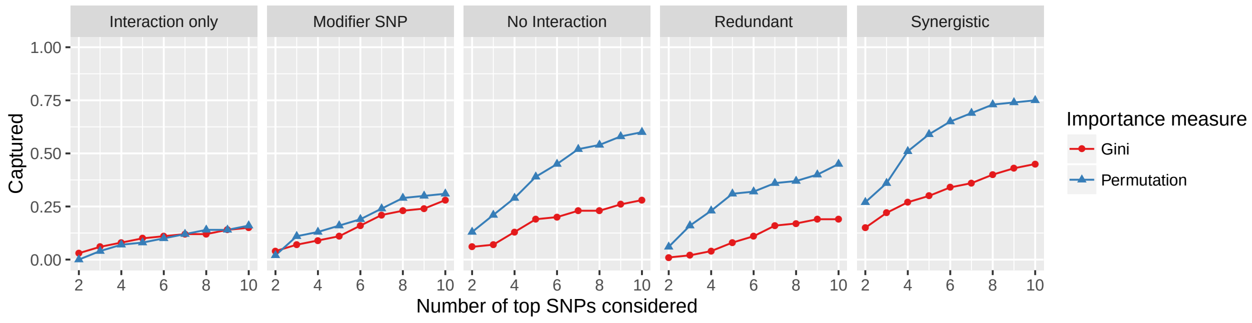

Figure S101: $\beta_I = 0.8, \beta_M = 0.4, mtry = 50$. Linkage disequilibrium.

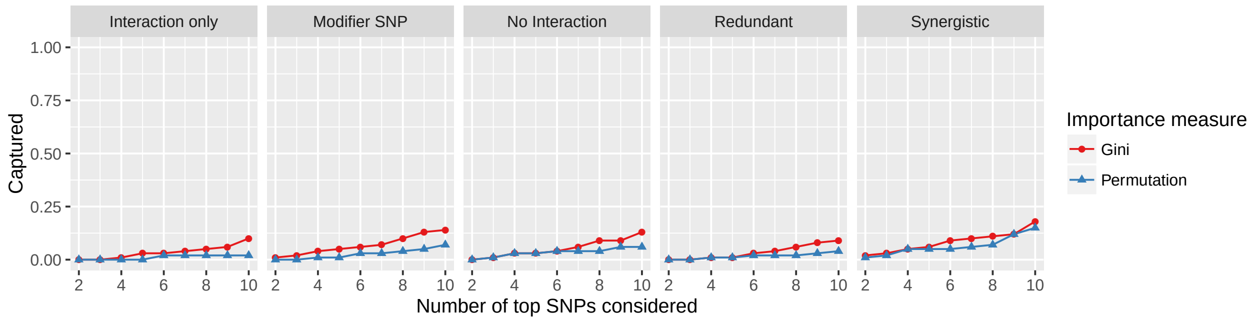

Figure S102: $\beta_I = 0.4, \beta_M = 0.8, mtry = 50$. Linkage disequilibrium.

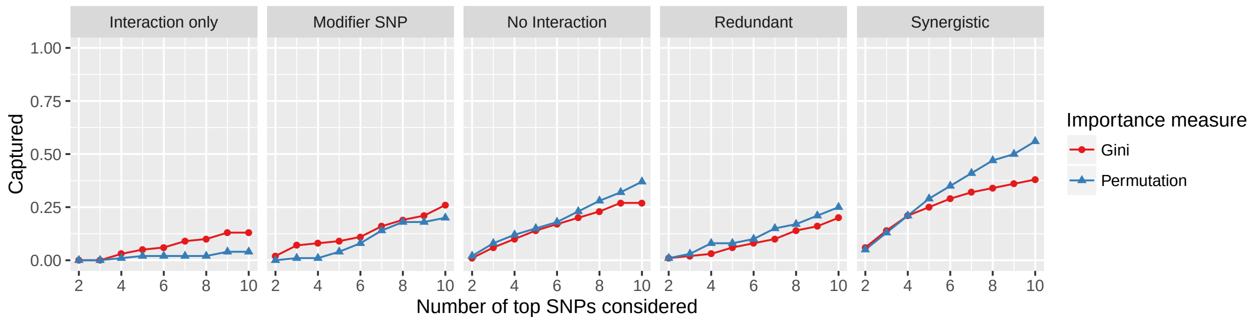

Figure S103: $\beta_I = 0.8, \beta_M = 0.8, mtry = 50$. Linkage disequilibrium.

Supplement for: *Do little interactions get lost in dark random forests?*

Part 7: Additional scenarios, pairwise variable importance measures

Marvin N. Wright, Andreas Ziegler, Inke R. König

1 Fewer marginal-only SNPs

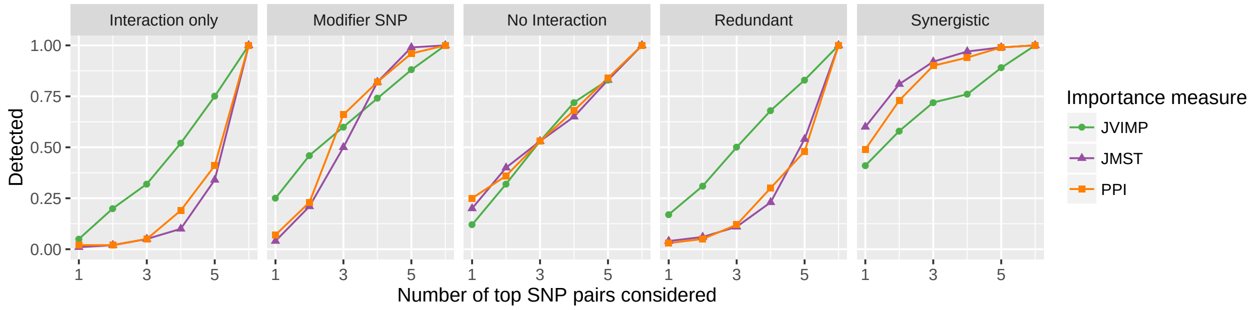

Figure S104: $\beta_I = 0.4, \beta_M = 0.4, MAF_I = 0.2, MAF_M = 0.2, mtry = 50$. 2 marginal-only SNPs.

2 More SNPs than samples

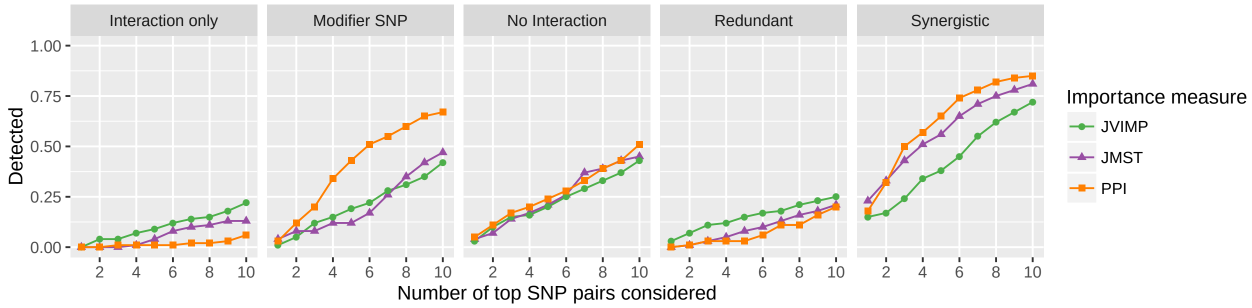

Figure S105: $\beta_I = 0.4, \beta_M = 0.4, MAF_I = 0.2, MAF_M = 0.2, mtry = 50, 2500$ SNPs.

3 Correlated SNPs

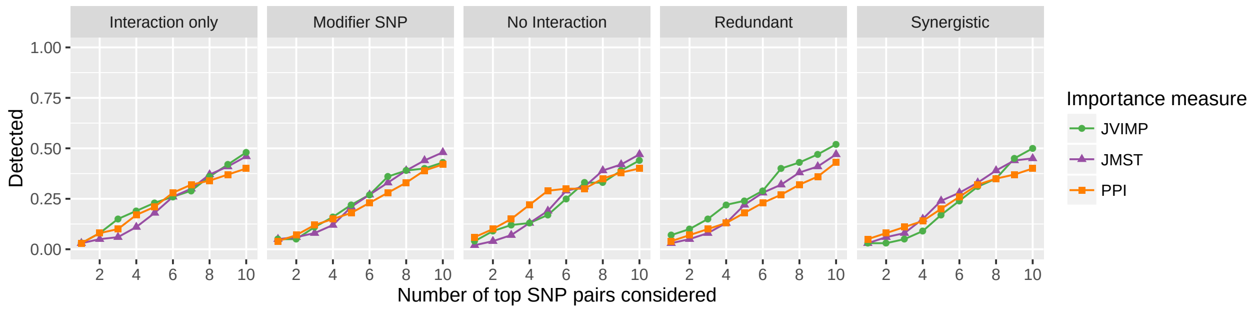

Figure S106: $\beta_I = 0, \beta_M = 0, mtry = 50$. Linkage disequilibrium.

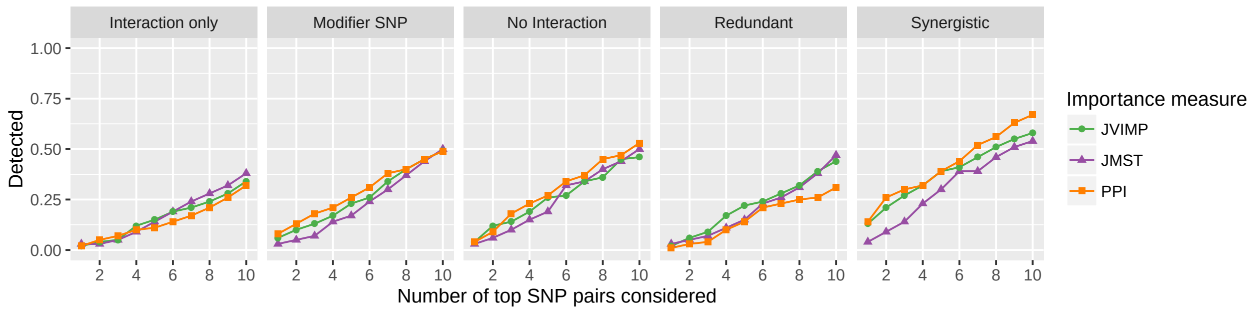

Figure S107: $\beta_I = 0.4, \beta_M = 0.4, mtry = 50$. Linkage disequilibrium.

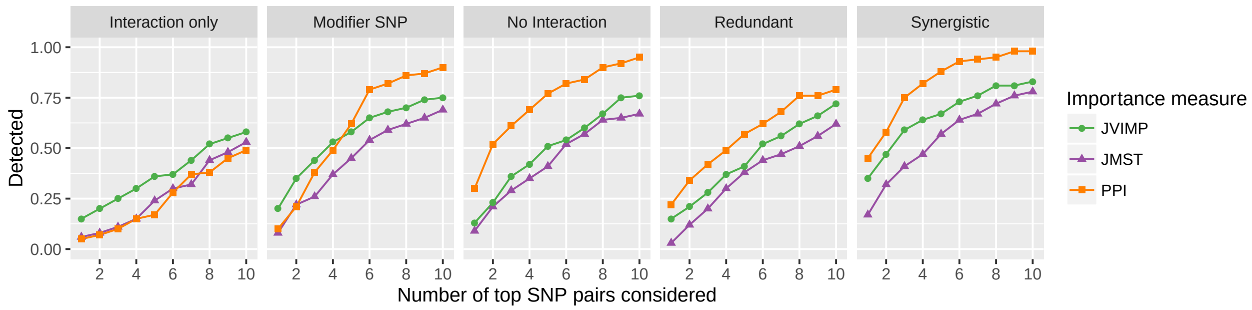

Figure S108: $\beta_I = 0.8, \beta_M = 0.4, mtry = 50$. Linkage disequilibrium.

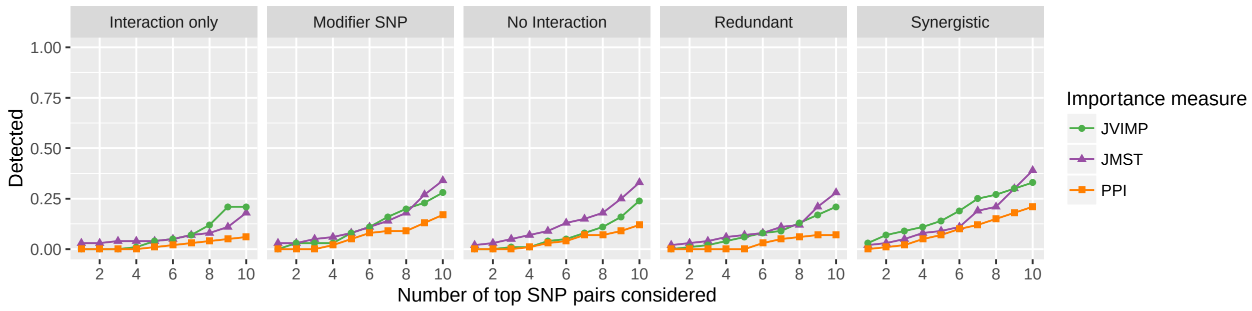

Figure S109: $\beta_I = 0.4, \beta_M = 0.8, mtry = 50$. Linkage disequilibrium.

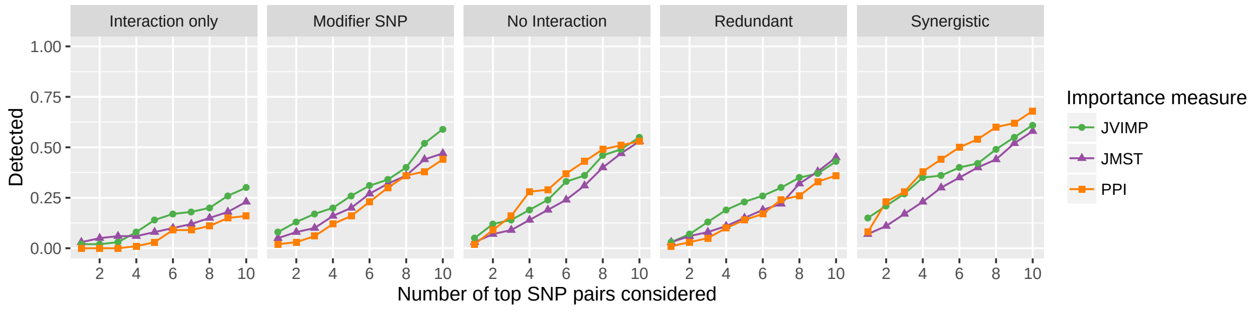

Figure S110: $\beta_I = 0.8, \beta_M = 0.8, mtry = 50$. Linkage disequilibrium.

Supplement for: *Do little interactions get lost in dark random forests?*

Part 8: Additional scenarios, ranks of marginal-only SNPs, single variable importance measures

Marvin N. Wright, Andreas Ziegler, Inke R. König

1 Fewer marginal-only SNPs

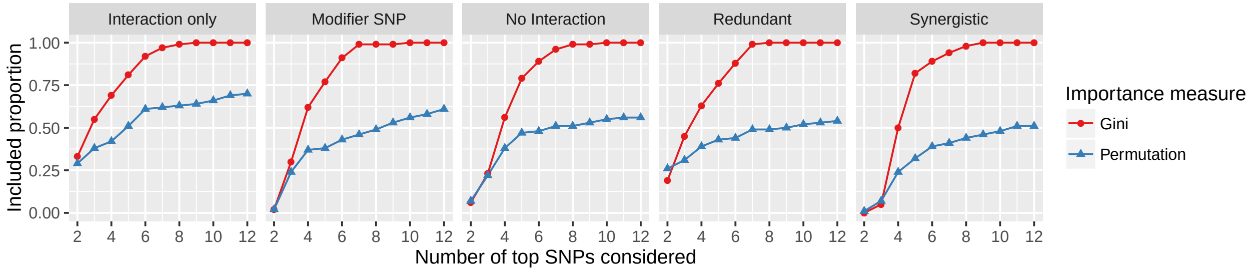

Figure S111: $\beta_I = 0.4, \beta_M = 0.4, MAF_I = 0.2, MAF_M = 0.2, mtry = 50$. 2 marginal-only SNPs.

2 More SNPs than samples

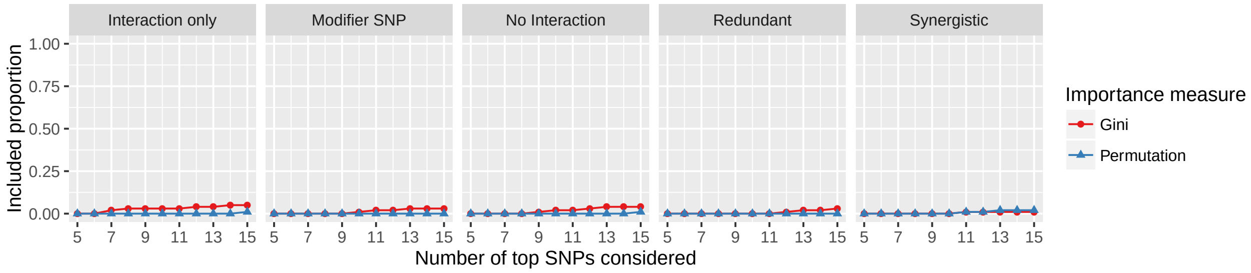

Figure S112: $\beta_I = 0.4, \beta_M = 0.4, MAF_I = 0.2, MAF_M = 0.2, mtry = 50, 2500$ SNPs.

3 Correlated SNPs

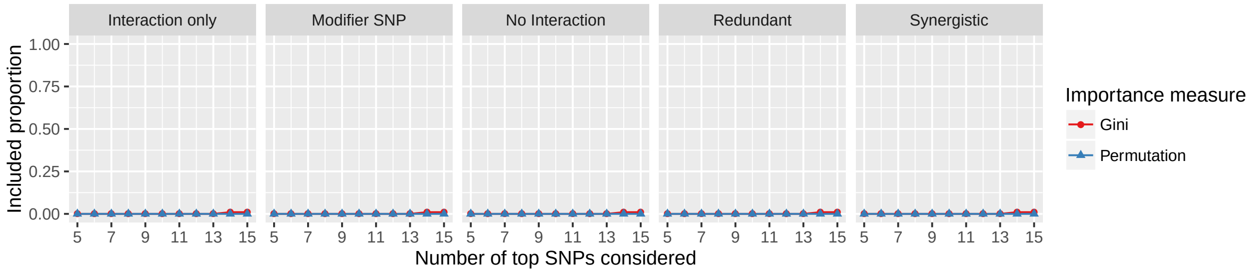

Figure S113: $\beta_I = 0, \beta_M = 0, mtry = 50$. Linkage disequilibrium.

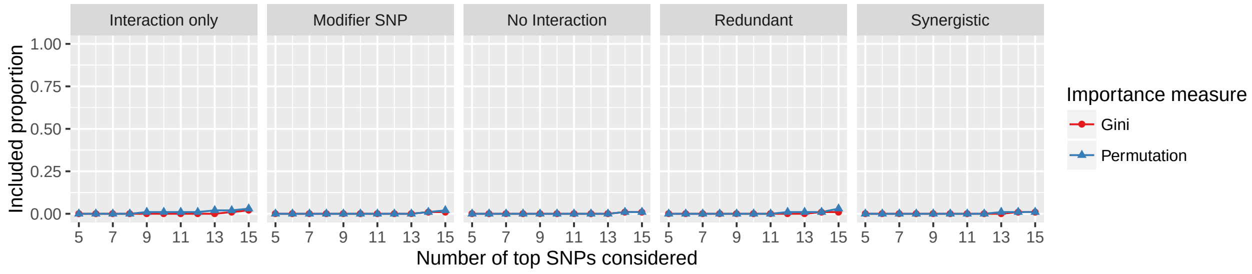

Figure S114: $\beta_I = 0.4, \beta_M = 0.4, mtry = 50$. Linkage disequilibrium.

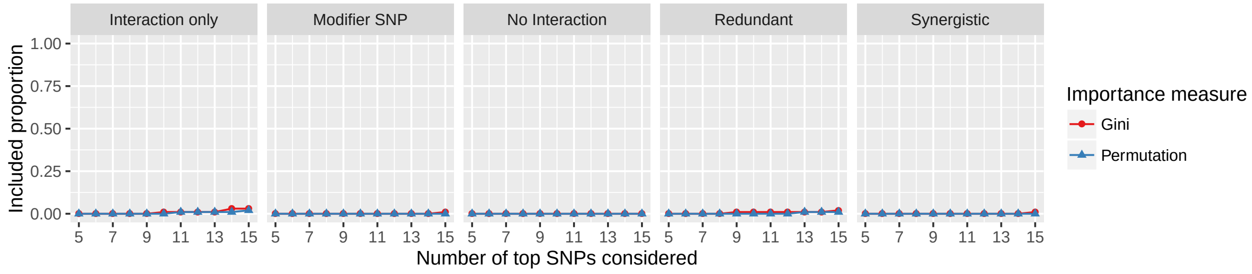

Figure S115: $\beta_I = 0.8, \beta_M = 0.4, mtry = 50$. Linkage disequilibrium.

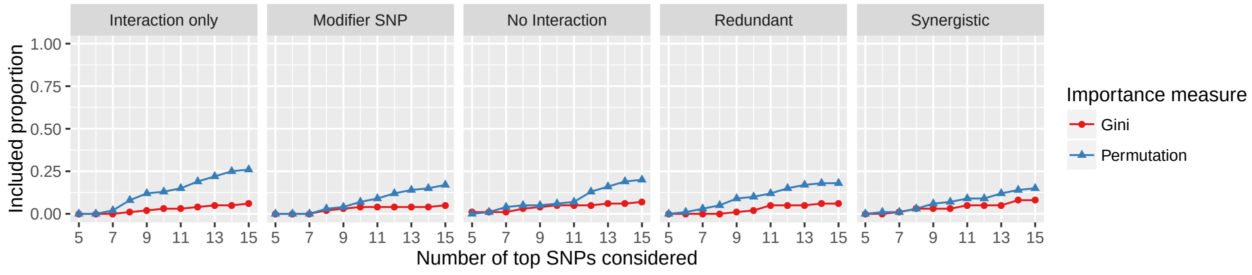

Figure S116: $\beta_I = 0.4, \beta_M = 0.8, mtry = 50$. Linkage disequilibrium.

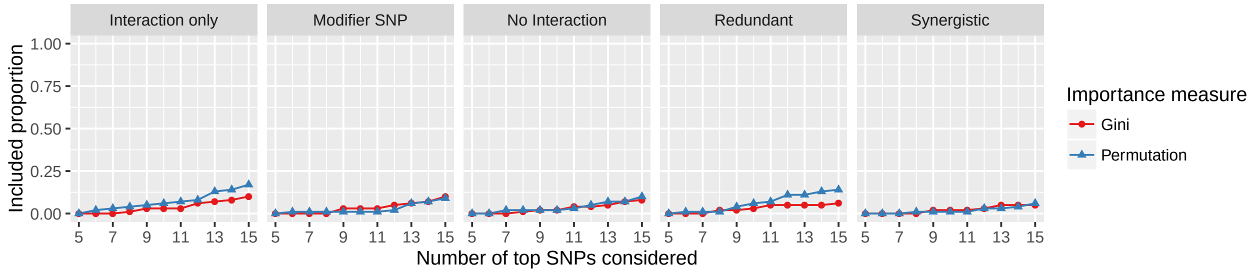

Figure S117: $\beta_I = 0.8, \beta_M = 0.8, mtry = 50$. Linkage disequilibrium.

Supplement for: *Do little interactions get lost in dark random forests?*

Part 9: Additional scenarios, ranks of marginal-only SNPs, pairwise variable importance measures

Marvin N. Wright, Andreas Ziegler, Inke R. König

1 Fewer marginal-only SNPs

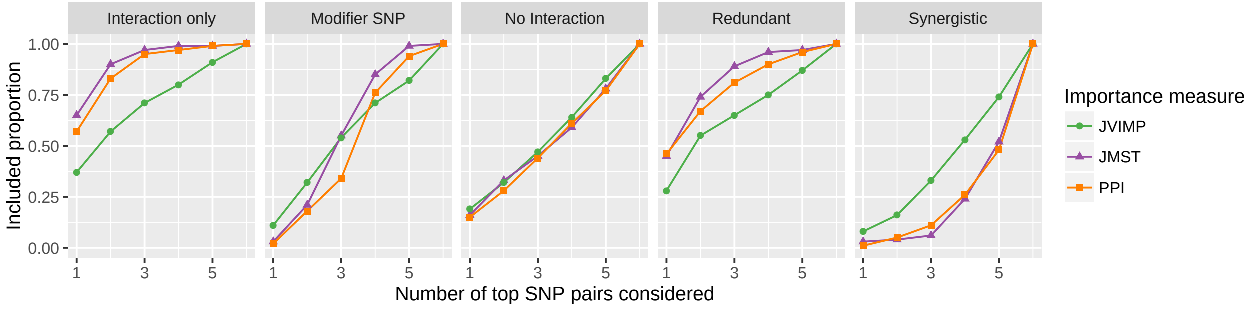

Figure S118: $\beta_I = 0.4, \beta_M = 0.4, MAF_I = 0.2, MAF_M = 0.2, mtry = 50$. 2 marginal-only SNPs.

2 More SNPs than samples

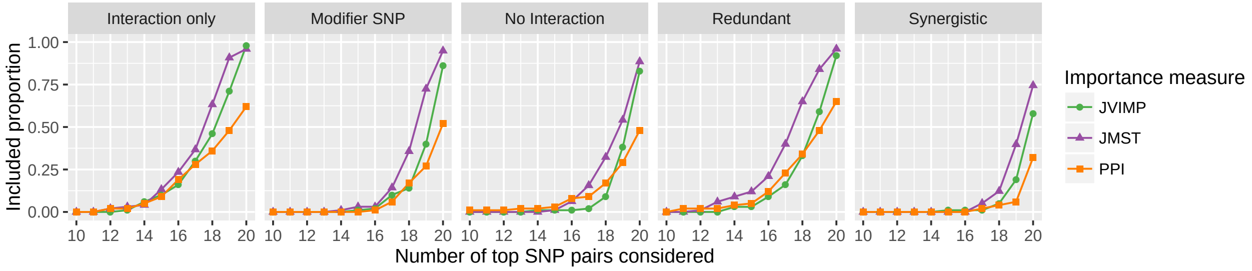

Figure S119: $\beta_I = 0.4, \beta_M = 0.4, MAF_I = 0.2, MAF_M = 0.2, mtry = 50, 2500$ SNPs.

3 Correlated SNPs

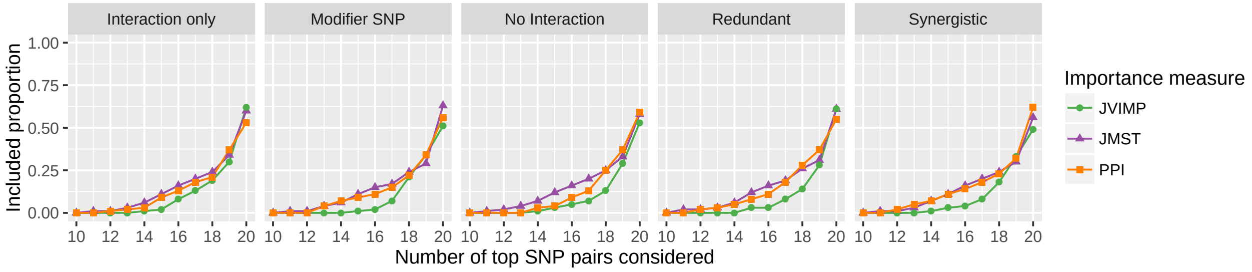

Figure S120: $\beta_I = 0, \beta_M = 0, mtry = 50$. Linkage disequilibrium.

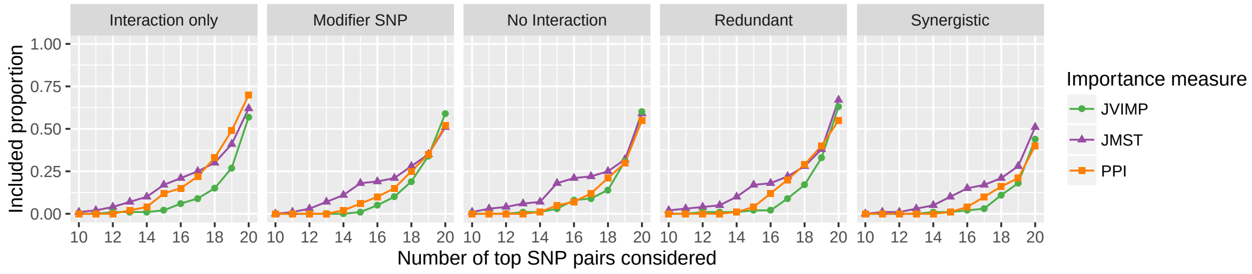

Figure S121: $\beta_I = 0.4, \beta_M = 0.4, mtry = 50$. Linkage disequilibrium.

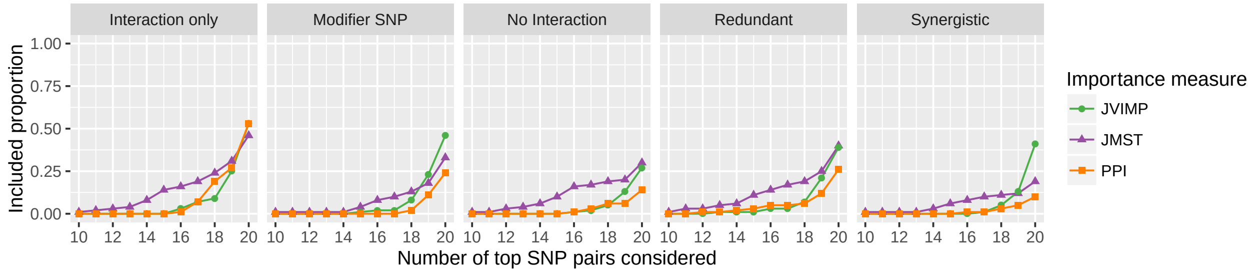

Figure S122: $\beta_I = 0.8, \beta_M = 0.4, mtry = 50$. Linkage disequilibrium.

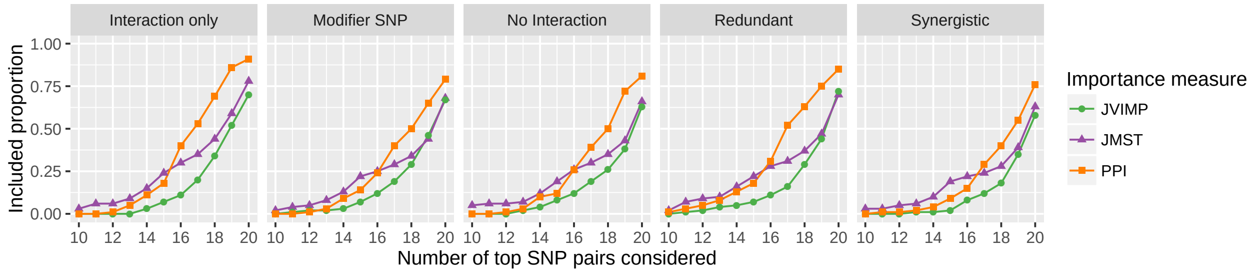

Figure S123: $\beta_I = 0.4, \beta_M = 0.8, mtry = 50$. Linkage disequilibrium.

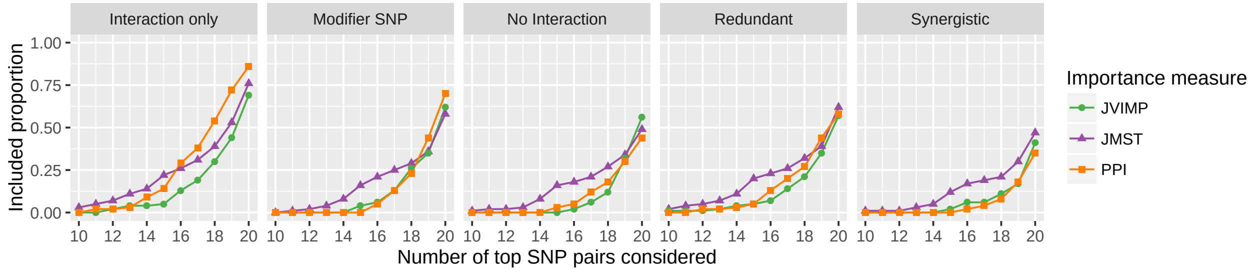

Figure S124: $\beta_I = 0.8, \beta_M = 0.8, mtry = 50$. Linkage disequilibrium.
